# Combined 3D-QSAR, molecular docking and dynamics simulations studies to model and design TTK inhibitors

**DOI:** 10.3389/fchem.2022.1003816

**Published:** 2022-11-02

**Authors:** Noureen Ashraf, Asnuzilawati Asari, Numan Yousaf, Matloob Ahmad, Mahmood Ahmed, Amir Faisal, Muhammad Saleem, Muhammad Muddassar

**Affiliations:** ^1^ Department of Biosciences, COMSATS University Islamabad, Islamabad, Pakistan; ^2^ Faculty of Science and Marine Environment, Universiti Malaysia Terengganu, Kuala Nerus, Terengganu, Malaysia; ^3^ Department of Chemistry, Government College University, Faisalabad, Pakistan; ^4^ Department of Chemistry, Division of Science and Technology, University of Education, Lahore, Pakistan; ^5^ Department of Biology, Syed Babar Ali School of Science and Engineering, Lahore University of Management Sciences, Lahore, Pakistan; ^6^ School of Biological Sciences, University of the Punjab, Lahore, Pakistan

**Keywords:** TTK inhibitors, molecular docking, 3D-QSAR, MD simulations, MMPBSA

## Abstract

Tyrosine threonine kinase (TTK) is the key component of the spindle assembly checkpoint (SAC) that ensures correct attachment of chromosomes to the mitotic spindle and thereby their precise segregation into daughter cells by phosphorylating specific substrate proteins. The overexpression of TTK has been associated with various human malignancies, including breast, colorectal and thyroid carcinomas. TTK has been validated as a target for drug development, and several TTK inhibitors have been discovered. In this study, ligand and structure-based alignment as well as various partial charge models were used to perform 3D-QSAR modelling on 1H-Pyrrolo[3,2-c] pyridine core containing reported inhibitors of TTK protein using the comparative molecular field analysis (CoMFA) and comparative molecular similarity indices analysis (CoMSIA) approaches to design better active compounds. Different statistical methods i.e., correlation coefficient of non-cross validation (r^2^), correlation coefficient of leave-one-out cross-validation (q^2^), Fisher’s test (F) and bootstrapping were used to validate the developed models. Out of several charge models and alignment-based approaches, Merck Molecular Force Field (MMFF94) charges using structure-based alignment yielded highly predictive CoMFA (q^2^ = 0.583, Predr^2^ = 0.751) and CoMSIA (q^2^ = 0.690, Predr^2^ = 0.767) models. The models exhibited that electrostatic, steric, HBA, HBD, and hydrophobic fields play a key role in structure activity relationship of these compounds. Using the contour maps information of the best predictive model, new compounds were designed and docked at the TTK active site to predict their plausible binding modes. The structural stability of the TTK complexes with new compounds was confirmed using MD simulations. The simulation studies revealed that all compounds formed stable complexes. Similarly, MM/PBSA method based free energy calculations showed that these compounds bind with reasonably good affinity to the TTK protein. Overall molecular modelling results suggest that newly designed compounds can act as lead compounds for the optimization of TTK inhibitors.

## Introduction

The dual specificity kinase TTK (Also known as monopolar spindle 1 or MPS1) is the core component of spindle assembly checkpoint that ensures accurate segregation of chromosomes during mitosis. TTK controls the bipolar attachment of chromosomes to spindle microtubules by regulating the spindle assembly checkpoint (Wei et al., 2005; Lindberg and Meijer, 2021; Xing et al., 2021). TTK is activated at the unattached kinetochores and recruits’ components of the mitotic checkpoint complex (MCC), thereby initiating SAC (Fisk and Winey, 2001; Stucke et al., 2002; Laufer et al., 2014; Huang et al., 2021). MCC hinders metaphase to anaphase transition by inhibiting the activation of Anaphase Promoting Complex/Cyclosome (APC/C) until all the kinetochores are correctly attached to the microtubules, which is prerequisite for accurate chromosome division (Wengner et al., 2016). TTK is composed of 857 amino acids with double lobed protein kinase structure. It comprises C terminal catalytic and activation loops having residues ranging from 515-794. The N terminal lobe (Glu 516-Met602) is smaller than the C terminal lobe (Asn606-Gln794) and has six beta sheets and one alpha helix. The larger C-terminal lobe, however, is more complex and consists of 2 beta sheets, 7 alpha helices together with the activation, catalytic and p + 1 loops. Both lobes join the hinge region through the amino acid residues Glu603 and Gly605. In its dormant state TTK is catalytically inactive as the activation loop is locked. However, phosphorylation at the activation loop enables the TTK to adopt an active conformation and elevate its catalytic activity (Wang et al., 2009). Besides its role in mitosis, it also plays a role in meiosis, cell transformation and cytokinesis (Maia et al., 2015; Sugimoto et al., 2017b). TTK overexpression is detected in many cancer types including, breast, hepatocellular and thyroid carcinomas (Maia et al., 2015; Lu and Ren, 2021). Overexpression of TTK is associated with high serum AFP (alpha-fetoprotein) levels, large tumor size, advanced TNM stage (tumor, nodes, and metastases), and distant metastases. Enhanced expression can also lead to centrosome duplication, genomic instability, mitotic check point failure, abrogated kinetochore attachment, incorrect spindle stress, and chromosomal misalignment (Liu and Winey, 2012; Liu et al., 2015a; Liu et al., 2015b; Sugimoto et al., 2017a). Due to its major role in mitotic checkpoint and overexpression in different malignancies, TTK is considered a potential anti-cancer drug target.

Studies involving RNA interference-mediated knockdown or chemical inhibition of TTK have validated it as a target for cancer therapeutics (Schmidt et al., 2005; Brough et al., 2011; Daniel et al., 2011). Several TTK inhibitors, therefore, have been discovered during the last decade. This includes NMS-P715, CCT251455, CFI-402257, BOS172722, S81694, BAY1161909 and BAY 1217389 with last five progressing to clinical evaluations (Chen et al., 2018). Similarly, some other small-molecule inhibitors i.e., Diaminopyridine, pyrrolopyrimidine and quinazolines containing compounds have shown low nano-molar activities with reasonably well growth inhibition of cell lines (Kusakabe et al., 2012; Bursavich et al., 2013).

In the current study Pyrrolo pyridine derivatives were used to develop 3D-QSAR models for designing of TTK inhibitors with improved activity. To the best of our knowledge so far, no QSAR modeling and docking simulations have been performed on this class of compounds. CoMFA and CoMSIA models were developed using different alignment schemes and charge models which were then validated using various statistical methods. The information derived from the models were exploited in designing of new compounds that are predicted to have better biological activities than the existing compounds in this class. The stability of binding modes and interactions of newly designed compounds with TTK protein were confirmed by MD Simulations.

## Materials and methods

### Data collection

Different reported inhibitors of TTK protein sharing similar scaffolds but different biological activities were retrieved from the literature (Naud et al., 2013). The IC50 values of all inhibitors were converted into pIC50 values. The 39 retrieved compounds were randomly divided into two groups: training (28 compounds) and test (11 compounds) datasets (Puzyn et al., 2011). The pIC50 values were used as dependent variable while CoMFA and CoMSIA descriptors were taken as independent variables (Balasubramanian et al., 2014).

### Structure preparation and alignment

The 2D structures of inhibitors were sketched by 2D builder tool of Maestro implemented in Schrödinger’s suite (Bhachoo and Beuming, 2017). The structures of all compounds were minimized by Conjugate gradient and Powell methods, while the partial charges were computed by Gasteiger Huckel (GH), Gasteiger Marsili (GM), Pullman, and MMFF94 charges (Sainy and Sharma, 2015; Shiri et al., 2016). The compound with the highest biological activity among all the inhibitors was selected as a template for ligand and structure-based conformer alignment of all compounds.

### CoMFA and CoMSIA field calculations

The CoMFA electrostatic and steric fields were calculated through SYBYL software using a 3D grid having a 2.0 Å spacing (Ghosh et al., 2021). A fixed energy value of 30 kcal/mol was set to avoid energy clashes. A carbon with sp^3^ hybridization and an atom with +1.0 charge were used as steric and electrostatic probes, respectively. A probe atom having a radius of 1.0 Å was used to calculate the CoMSIA fields. The attenuation factor (α) with a default value 0.3 was used to calculate the distance dependent similarities. The Eq. 1 was used to calculate the indices. All computations were carried out in the same way as the CoMFA analysis (Hu et al., 2009; Li et al., 2017).

$$A_{F,K}^{q}\left(j\right)=\sum \omega probe,k\omega ike^{{-ar}^{2}}iq$$ (1)

A^q^ = similarity indexK = physiochemical properties of CoMFA fields descriptorsωprobe = the probe atomi = summation index of molecule jωik = observed value k of a specific property of the atom ir = atomic radiusThe efficiency of SAR model was determined by Partial Least Square regression. The CoMFA and CoMSIA descriptors were selected as dependent variables while IC50 value was selected as an independent variable in PLS regression (Tahir et al., 2018). The cross validation using the leave-one-out method was used to select the best model that had high prediction power. The cross-validation (q^2^) analysis is defined by Eq. 2.

$$q^{2}=1-\frac{\sum_{y}{\left(y_{pred}-y_{obs}\right)}^{2}}{\sum_{y}{\left(y_{obs}-y_{mean}\right)}^{2}}$$ (2)

y_pred_ = predicted valuesy_obs_ = experimental valuesy_mean_ = mean values

For non-cross validation, the column filtering was set to 2.0. Standard error estimation (SEE) values were also calculated along cross and non-cross validation. To evaluate the effectiveness of the generated models, bootstrapping was used up to 100 runs. Predictive *r* ^2^ was used to express the predictive ability of the developed models, that was based on the test set compounds. The predictive *r* ^2^ was calculated using Eq. 3.

$$r_{pred}^{2}=\frac{\left(SD-PRESS\right)}{SD}$$ (3)

SD = sum of squared deviations between pIC50 values of the test set and mean pIC50 values of the training setPRESS = sum of squared deviations between the test molecules observed and expected activities

### Designing of new compounds

Based on the information obtained from the contour maps of best predictive CoMFA and CoMSIA models, ten new compounds were designed by substitution of specific electrostatic, steric, hydrophobic, hydrogen bond donor, and hydrogen bond acceptor groups to enhance their inhibitory activities against TTK protein. The newly designed compounds belong to the synthetic class of compounds and their biological activities were predicted using the best predictive models (Lorca et al., 2018; Ghosh et al., 2021).

### Molecular docking

The co-crystal structure of TTK (PDB ID: 4C4J) was prepared by protein preparation wizard implemented in Maestro. The receptor was preprocessed by adding hydrogens, removing water, adding charges and fixing residues side chain atoms. The unnecessary ligands and chains were removed while the tautomeric states were generated at pH 7.0. The structure of the receptor was further optimized and minimized by OPLS_2005 forcefield [34]. The grid was generated by selecting the co-crystal ligand to perform site-specific docking. To soften the potential of non-polar sections of the receptor, the van der Waals radii of the receptor atom were scaled to 1.0 and the partial charge cutoff value was set to 0.25. The values for the X, Y, and Z coordinates were 0.8, 17.52, and 45.37 respectively. After grid generation, newly designed compounds were prepared by LigPrep tool of Maestro prior to docking [35]. Different ionization states were generated at pH 7 by using Epik [35]. The stereoisomers of compounds with specified chirality were generated by using OPLS_2005 forcefield. The prepared ligands were then docked to the prepared receptor by using the Glide docking tool and the binding poses were analyzed based on the glide gscore.

### MD simulations

The binding poses of each compound were used to make complexes with the TTK protein. The stability of each protein-ligand complex was estimated by running MD simulation using NAMD (Acun et al., 2018). All the complexes were prepared by using LeaP module of AMBER21 tools (Case et al., 2021). The parameters of the ligands were generated by antechamber program by semi-empirical calculation. The PDB4amber module was used to convert the amino acid residues to amber format. The forcefield parameters for protein and ligands were AMBER ff14SB force field and general amber forcefield, respectively (Duan et al., 2003). The parameters of ligands and receptor were connected by tleap program. All the complexes were solvated in a water box of size 10 Å using TIP3P water model. To neutralize the system, counter ions Na^+^ and Cl^−^ were added by using LeaP. The systems were minimized by conjugating gradient and steepest descent method for 10,000 times. The water equilibration was done for 5,000 steps, followed by the three-temperature equilibration from 0 to 200 K, 200–250 K, and 250–300 K for 5,000 steps. After equilibration of the system at different temperatures, the production of systems was run for 25 ns with constant temperature 310 K and pressure of 1 atm using NPT ensemble. The trajectories of all the systems were analyzed to get RMSD, RMSF, Radius of gyration, SASA, PCA, by using VMD tcl commands, CPPTRAJ (Roe et al., 2013) and R package.

### Binding free energy calculation

The binding free energy of the system was calculated by using molecular mechanics-based scoring methods MM/PBSA (Sun et al., 2014). The calculations were based on a total of 300 snapshots of the complex, taken at 2 ps interval from the last 2 ns stable MD trajectories. The binding free energy was determined as the difference between the total free energy (ΔG_com_) of the ligand-receptor complex and the sum of free energy of individual receptors (ΔG_pro_) and ligand (ΔG_lig_) using the equation provided below:

$${\Delta G}_{bind}=\Delta H-T\Delta S={\Delta G}_{com}-\left[{\Delta G}_{pro}+{\Delta G}_{lig}\right]$$

The ΔG for the complex, receptor and ligand can be calculated by the following equation:

$$\Delta G={\Delta E}_{MM}+{\Delta G}_{sol}-T\Delta S$$

ΔE_MM_ = Molecular Mechanics EnergyΔG_sol_ = Solvation Free EnergyTΔS = Entropy at given Temperature

### ADMET analysis

The physicochemical properties i.e., molecular weight, Hydrogen bond donors and acceptors along with the ADMET properties of the newly designed compounds were predicted by QikProp tool of Maestro (Koç et al., 2021).

## Results and discussion

**FIGURE 1:**
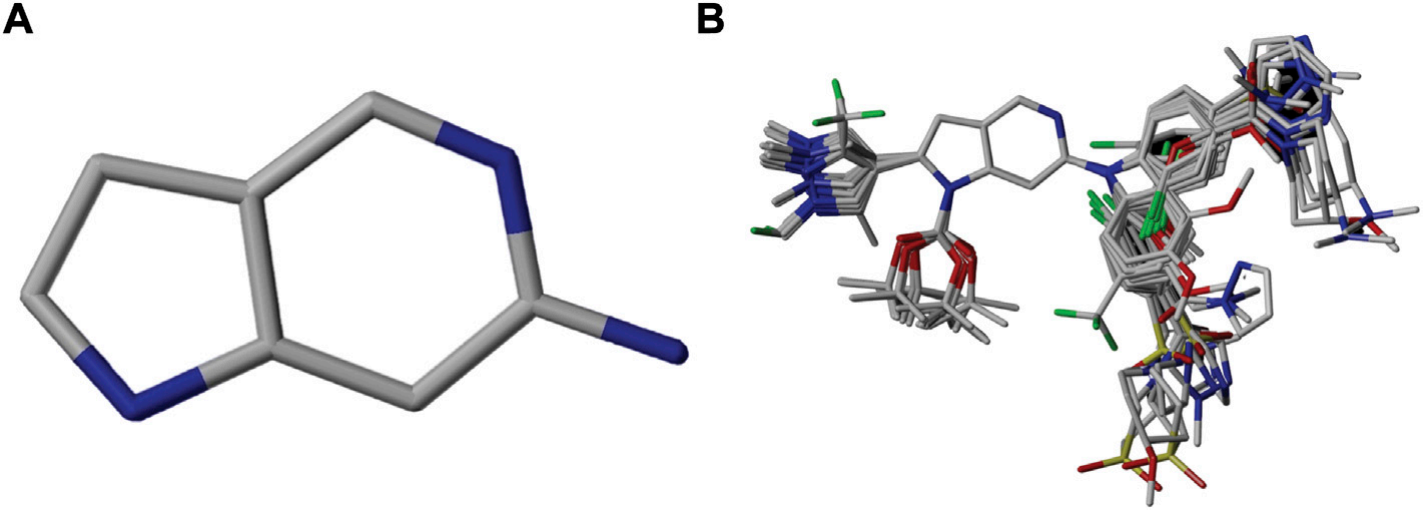
Alignment of dataset compounds: **(A)** Common Substructure and **(B)** Structure-based alignment of docked compounds.

An essential stage in ‘3D-QSAR’ is the systematized assortment of compounds and their division into training and test datasets. Compounds and their biological activities in terms of pIC50 values are mentioned in Supplementary Table S1. They were classified into two categories with respect to their activity range from high to low while maintaining structural variations. All the selected compounds possess a common sub-structure 1H-Pyrrolo[3,2-c] pyridine as shown in Figure 1A. These compounds have mainly hydrophobic (halogens Cl and Br) and hydrophilic substituents (amine and amides) attached to the core scaffold 1H-Pyrrolo[3,2-c] pyridine. Hydrogen bond donors like NH and OH, hydrogen bond acceptors like N, O and F and steric groups like CH3 and Cl have been attached to enhance the activities of the compounds. By changing the substituent at main scaffold, the activity of the compounds predicted by the developed models, was affected. The quality of the models is affected by multiple factors like the conformation of the molecules and their assigned partial charges (Muddassar et al., 2009; Wang et al., 2015). Therefore, different conformations of dataset molecules using ligand and structure-based approaches were generated along with different charge models i.e., GH, GM, MMFF. For structural alignment, compounds were aligned on a common sub-structure to get the best predictive “CoMFA” and “CoMSIA” models (Figure 1).

**FIGURE 2:**
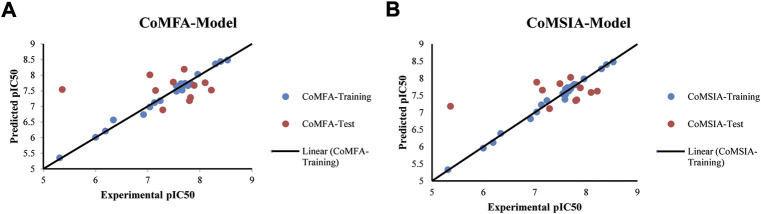
Correlation plots between experimental and predicted biological activities **(A)** From CoMFA Model **(B)** From CoMSIA Model.

The best models were obtained with Merck Molecular Force Field charges using structure-based conformation alignment as shown in Figure 1B. The correlation coefficient q^2^ of leave-one-out cross validation for CoMFA fields was 0.589, with 3 optimum number of components, the standard error of estimation was 0.088, non-cross validated coefficient (r^2^ncv) = 0.902, F-value = 73.624 and r^2^pred = 0.751 as mentioned in Table 1. The electrostatic and steric fields contributed 68.3% and 31.7% respectively to the model. However, ligand-based conformations yielded the poor predictive models. Powell method generated conformation with MMFF charges produced best CoMFA model with q^2^ = 0.268 value for 3 optimum number of components (other data shown in Supplementary Table S2). Similarly Conjugate Gradient conformation method with MMFF charges using 2 optimum number of components yielded q^2^ = 0.191 value for steric and electrostatic fields (Supplementary Table S3). In the ligand-based alignment technique, the effects of different charges on the models are shown in Supplementary Table S3. The reasons for superior performance of one charge method over the other in “CoMFA” and “CoMSIA” predictive models are still unknown, as the literature shows variable performance of these charge models on compounds targeting different proteins. As for as COMSIA models are concerned, structure-based alignment with Merck Molecular Force Field charges produced q^2^ = 0.690 with N = 3, SEE = 0.109, F-value = 108.296, r^2^ncv = 0.931, and r^2^pred = 0.767 shown in Table 1. The CoMSIA fields like steric, electrostatic, hydrophobic, hydrogen bond donor and acceptor contributions were 12.9%, 23.1%, 25.1%, 17.7% and 21.2% respectively. The results exhibited that electrostatic and hydrophobic interactions and hydrogen bond donors played major role in CoMSIA model. In CoMSIA modeling GH, GM, PM, and MMFF94 charges did not significantly influence the quality of models. Using the best predictive CoMFA and CoMSIA models, the biological activities of the training and test dataset compounds were predicted as shown in Figures 2A–B , respectively. The scattered plots show that the predicted values are similar to the experimental values except one compound (Outlier). Outliers can occur as a result of incorrectly measured inhibitory concentrations, variable binding confirmations, or major physicochemical variances. Similarly, external validation (higher r^2^pred values of test set compounds) of both models shows their highly predictive nature. Internal validations such as r^2^ncv, F-values, and r^2^bs values revealed their reliability and precision to design and improve new compounds. As any individual field can influence the quality of the model, therefore models with good statistical significance were used to design new compounds for improved activity as shown in Table 2.

**TABLE 1:** Statistical parameters of structure based CoMFA and CoMSIA models with different charge schemes.

	Gasteiger Huckel charges (GH)		Gasteiger Marsili Charges (GM)		Pullman charges (PM)		Merck molecular force field (MMFF94)	
Parameters	CoMFA	CoMSIA	CoMFA	CoMSIA	CoMFA	CoMSIA	CoMFA	CoMSIA
N	3	3	3	3	3	3	3	3
q^2^	0.583	0.705	0.584	0.665	0.575	0.663	0.589	0.690
r^2^(NoV)	0.891	0.946	0.888	0.918	0.893	0.950	0.902	0.931
SEE	0.090	0.075	0.090	0.068	0.093	0.087	0.088	0.109
F	65.535	139.223	63.112	89.516	66.594	152.924	73.624	108.296
Pred (r^2)^	0.638	0.814	0.767	0.804	0.619	0.721	0.751	0.767
^r2^bs	0.928	0.959	0.913	0.937	0.942	0.930	0.919	0.941
SD_bs_	0.215	0.168	0.216	0.179	0.206	0.201	0.216	0.187
Fields contribution								
Steric (S)	0.714	0.126	0.724	0.139	0.670	0.128	0.683	0.129
Electrostatic(E)	0.286	0.227	0.276	0.192	0.330	0.216	0.317	0.231
Hydrophobic (H)	-----	0.263	-----	0.258	-----	0.272	-----	0.251
Donor (D)	-----	0.171	-----	0.166	-----	0.172	-----	0.177
Acceptor (A)	-----	0.213	-----	0.245	-----	0.212	-----	0.212

N, “Optimal number of components; q^2^, cross-validated correlation coefficient; r^2^, determination coefficient; r^2^ nov, non-cross validated correlation coefficient; SEE, standard error of estimate; F, Fischer’s test F-value; Pred-r^2^, predictive r^2^ for test set compounds; r^2^ bs, r^2^ obtained after 100 bootstrapping runs; and SD_bs_, bootstrapping standard deviation.

**TABLE 2:** Comparison of parent and modified compounds activities.

Parent compounds		Actual pIC50	Modified compounds		Predicted-pIC50
9	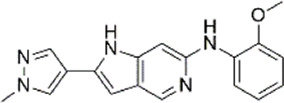	6.92	NDC1	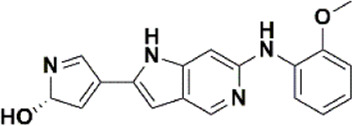	7.04
19	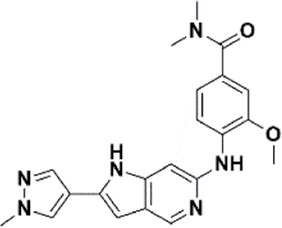	7.63	NDC2	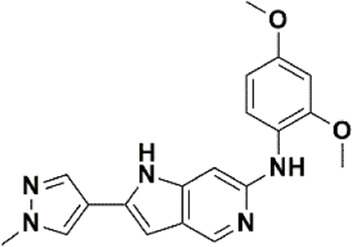	6.96
9	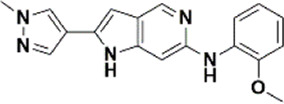	6.92	NDC3	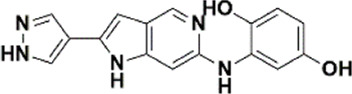	7.36
2	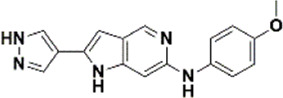	7.29	NDC4	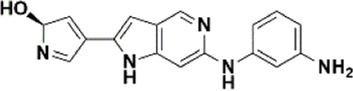	7.10
15	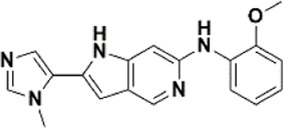	6.19	NDC5	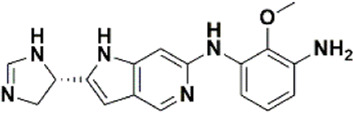	7.24
16	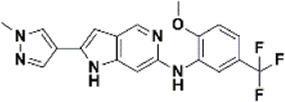	5.36	NDC6	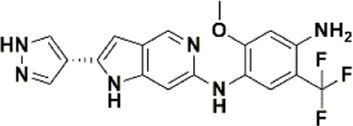	7.13
10	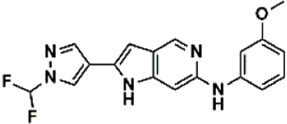	6.34	NDC7	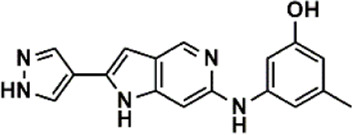	7.13
26	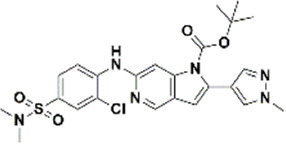	7.03	NDC8	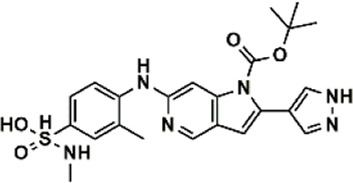	7.913
37	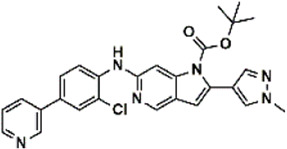	7.56	NDC9	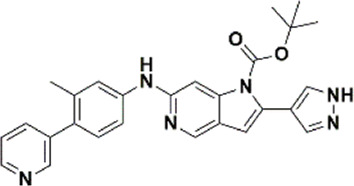	8.062
39	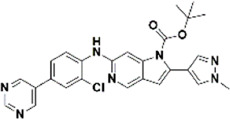	7.68	NDC10	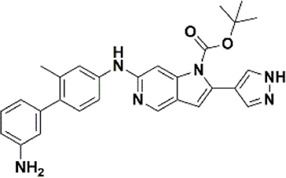	8.063

### CoMFA contour maps

**FIGURE 3:**
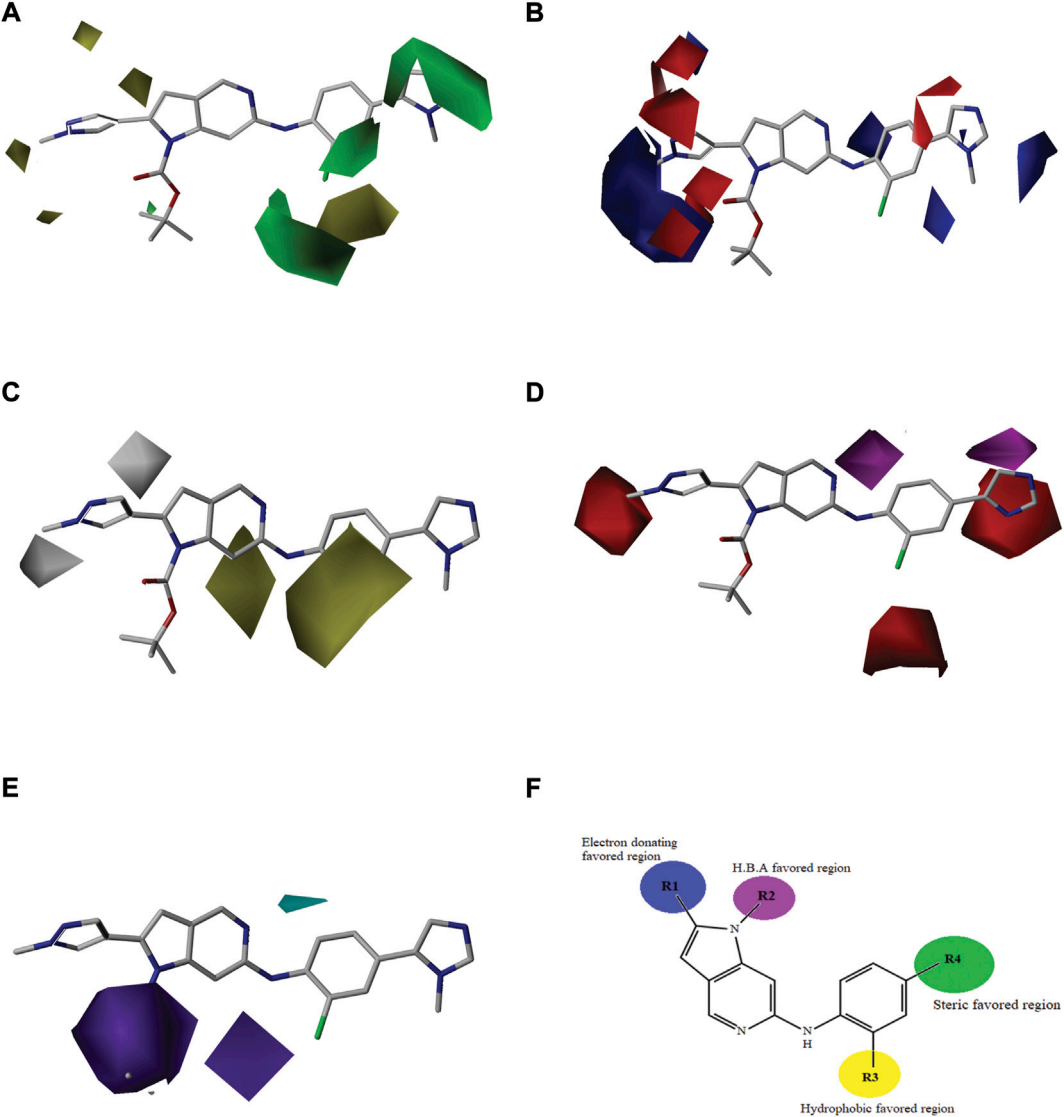
“Structure-based model of the most active compound represented through contour maps (36). **(A)** Contour maps of CoMFA steric field; **(B)** Contour maps of CoMFA electrostatic fields; **(C)** Contour maps of CoMSIA hydrophobic field; **(D)** Contour maps of CoMSIA hydrogen bond acceptor fields; **(E)** Contour maps of CoMSIA hydrogen bond donor fields; **(F)** Structure activity relationship representation of contour groups.”

Contour maps of the best predictive models were generated on the most active compound, and then this 3D information was exploited to create new compounds predicted to have improved biological activities. The contour maps of CoMFA fields for the best model are shown in Figures 3A,B. The steric contour maps are shown in Figure 3A, the green contour denotes the favored area for bulky group substitution, whereas the yellow contour shows the disfavored area for bulky group substitutions. The replacement of bulky groups at R4 position will increase the activity of compounds. For example, compound 27 (pIC50 = 8.1) with azetidine amide at R4 is predicted to be more active than compound 17 (pIC50 = 7.13) which has nothing at same position. Figure 3B shows the electrostatic field contour maps. The red and blue contours represent the effect of the electrostatic field on the biological activity of compounds. The large blue contour near R1 position shows that the substitution of electron donating group will increase the activity of compound that’s why the activity of compound 1 (pIC50 = 7.60) having electron donating nitrogen at R1, is better than compound 10 (pIC50 = 6.34) that has electron with drawing difluromethyl at the same position. Similarly, the red contour near the R2 indicates that the replacement with electron withdrawing group will increase the bioactivity of the compounds. These observations are in agreement with previously published results (Vaidya et al., 2017).

### CoMSIA contour maps

The contour maps of CoMFA and CoMSIA showed the similarity in steric and electrostatic fields. The remaining fields of CoMSIA i.e., hydrophobic, HBA and HBD are shown in Figures 3C,D,E. Figure 3C shows the hydrophobic contour, where yellow contour at the R3 position indicates that the substitution of the hydrophobic group is favorable to increase the activity while white contour near R1 position shows that the activity can be increased by replacing the hydrophilic group at this position. Therefore, compounds 22, 23, 28-30 and 33-39 with hydrophobic groups at the R3 position showed significant predicted biological activities. Figure 3D indicates the hydrogen bond acceptors contour. Magenta contour shows the area which is favorable for hydrogen bond acceptor group substitution while red region is favorable for hydrogen bond donor group substitution to increase the activity of compounds. Similarly, the purple contour in Figure 5E shows the disfavored area for hydrogen bond donor group substitution. So, the substitution of hydrogen bond donor groups at R1 and R4 position will increase the biological activity of compounds, while the substitution of hydrogen bond acceptor groups at R2 position will increase the activity. In the contour maps, hydrogen bond acceptors and donors shared 80% for favored regions while 20% for unfavored regions to increase the biological activity of compounds. The structure-activity relationship diagram (Figure 3F) was obtained from the CoMFA and CoMSIA contour maps. In order to design new compounds with better biological activities, the regions R1, R2, R3, and R4 are favorable for substitutions of electron donating groups, hydrogen bond acceptor groups, hydrophobic groups and bulky groups, respectively. In order to design new compounds, different modifications in the parent structures have been introduced based on the best CoMFA and CoMSIA models contours. For example, pyrazole ring of compound 9 showed favorable region for electron donating groups, so by replacing the methyl group with hydroxyl group, new compounds showed better predicted activity than parent compound. Similarly, a hydroxyl group was added to the compound 2 to get a new molecule with better activity. All new compounds were designed by adding specific groups at the favorable electron donating, hydrogen bond acceptor and steric group regions for better activities.

### Binding mode elucidation of newly designed compounds

**FIGURE 4:**
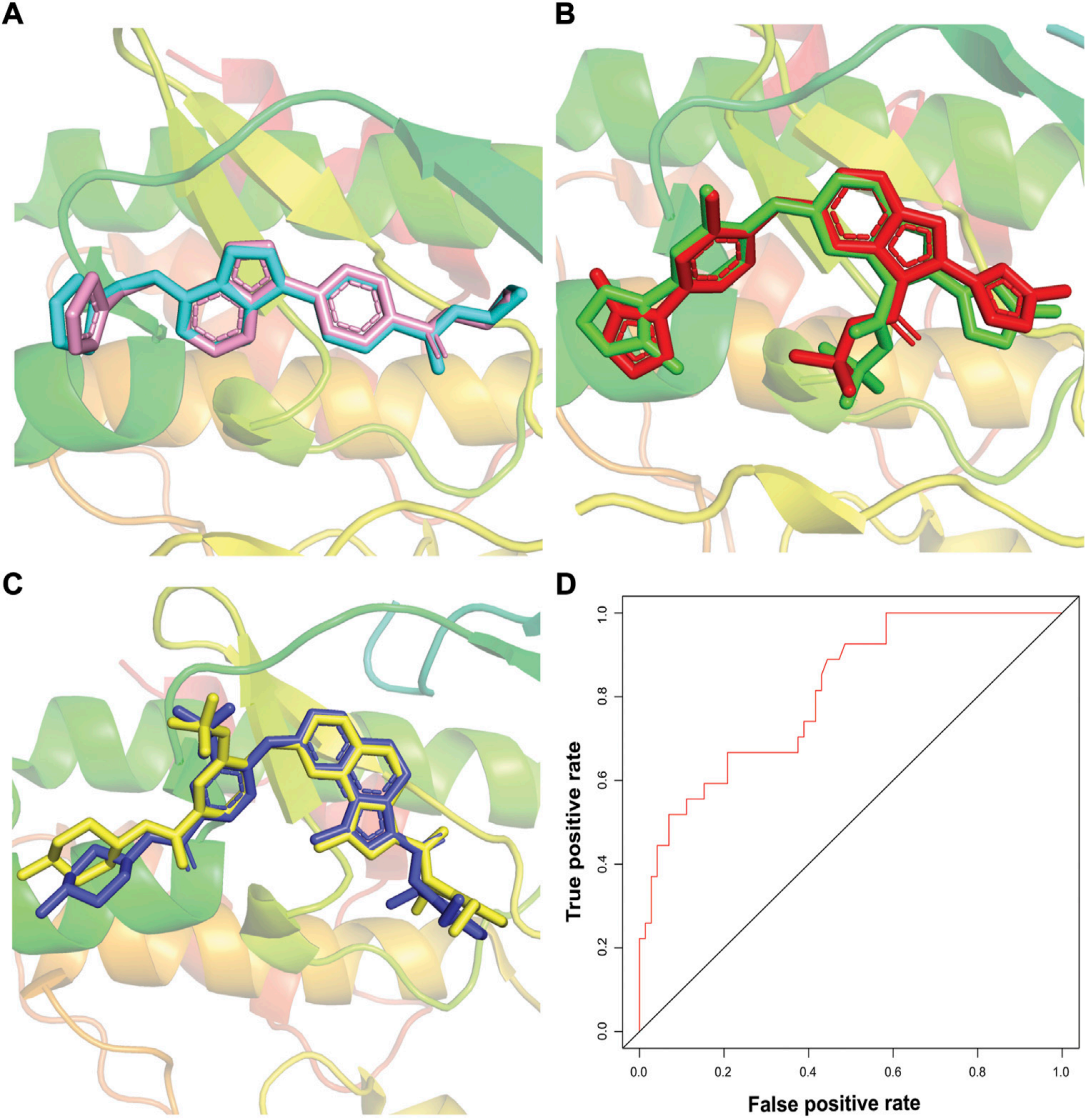
Docking protocol validation studies. The redocking of co-crystal ligands of three TTK X-rays crystal structures, **(A)** redocked pose of ligand in PDB ID: 3WZK is shown in cyan sticks, **(B)** redocked pose of ligand in PDB ID: 4C4J isshown in green sticks, **(C)** redocked pose of ligand in PDB ID: 5AP7 is shown in yellow sticks. The RMSD of redocked poses was less than 1Å. **(D)** The estimation of docking accuracy by AUC curve with a value of 0.80.

**FIGURE 5:**
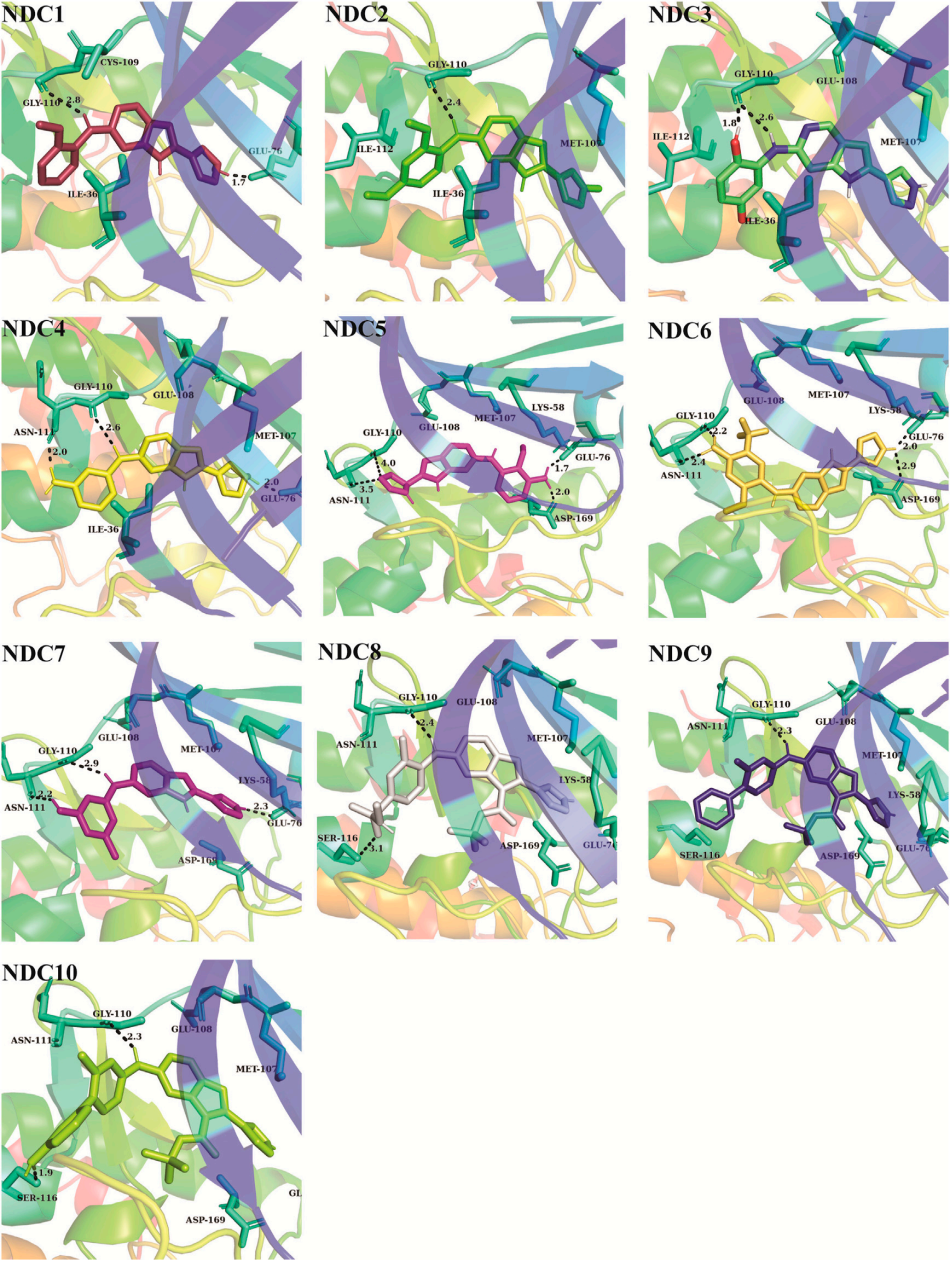
The binding interactions of newly designed compounds with the key residues of TTK binding pocket. The hydrogen bonds are denoted with black dash lines. The distance between the compounds and binding site residues is measured in Å.

Newly designed compounds NDC1-10 were docked into the active site of TTK protein to identify their plausible binding modes. Prior to the docking of newly designed compounds, the glide docking protocol was validated by calculating the RMSD of redocked poses of co-crystal ligands (Muddassar et al., 2010). The co-crystal ligands were extracted from the co-crystal structures (PDB IDs: 3WZK, 4C4J, 5AP7) and docked again at the same binding position where cocrystal ligands were making the hydrogen bonding interactions with the hinge region residues. The docked pose was then aligned on the native ligand which showed identical interaction with <1.0 Å deviation from original pose. The redocking of representative co-crystal ligands can be observed in Figures 4A–C. Moreover, the accuracy of glide tool was estimated by area under curve studies. A decoy dataset of 917 compounds was used along with active compounds of TTK. The AUC curve value of 0.80 showed that the true positive rate was higher than the false positive results produced by the glide scoring scheme as shown in Figure 4D. After validation of docking method, newly designed compounds were docked in the active site of TTK protein. All compounds showed good binding affinities in terms of glide scores given in Supplementary Table S4. The binding interactions of the newly designed compounds were analyzed and it was observed that all compounds were making hydrogen bonds with the hinge region residues especially Gly110. The other interacting residues were Ile36, Lys58, Glu76, Met107, Cys109, Asn111, Ile112, Ser116, Lys120, Asp169, Met176 and Pro178. In case of hydrogen bonding, NDC1 made two hydrogen bonds with Glu76 and Gly110 with a distance of 1.7 and 2.8 Å respectively. NDC2 made one hydrogen bond with Gly110, while NDC3 was making two hydrogen bonds with Gly110. The same bonding pattern was observed in all complexes i.e., hydrogen bonding with Gly110, Glu76. Moreover, the residues Ile36, Ile112, Met105, Met176 and Pro178 were involved in hydrophobic interactions with the newly designed compounds. The hydrogen bonds and the distances between the ligands and key residues are given in Figure 5.

### MD simulation analysis

**FIGURE 6:**
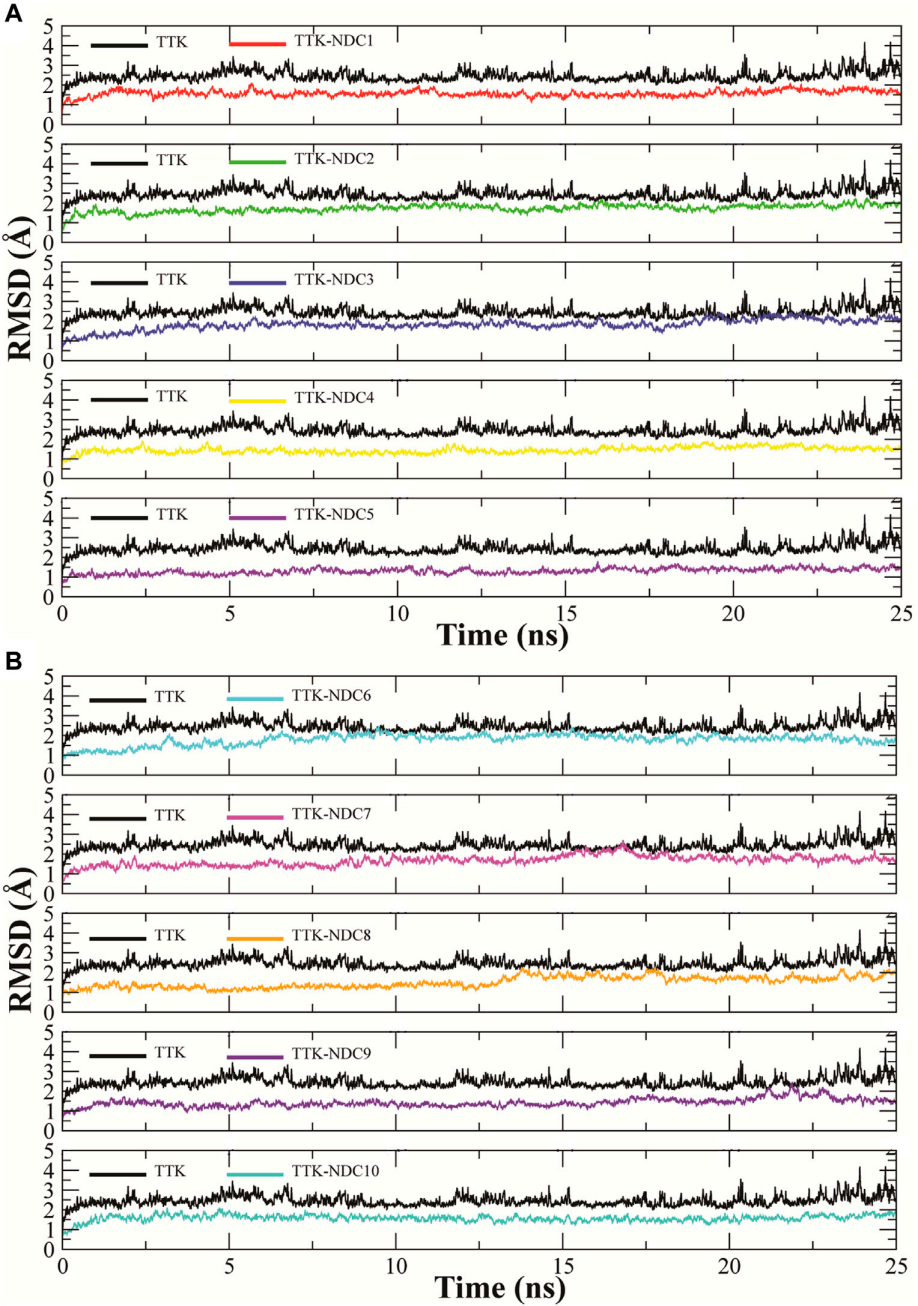
Root Mean Square Deviations in backbone of TTK bound to newly designed compounds; **(A)**TTK-NDC1 (red), TTK-NDC2 (green), TTK-NDC3 (blue), TTK-NDC4 (yellow), TTK-NDC5 (violet) **(B)**TTK-NDC6 (cyan), TTK-NDC7 (magenta), TTK-NDC8 (orange), TTK-NDC9 (indigo) and TTK-NDC10 (turquoise).

MD simulations were carried out to estimate the steady nature and stability of the protein and ligand complexes. The protein-ligand complex stability was estimated by the Root Mean Square Deviation (RMSD) of the complexes in 25 ns long simulation. The RMSD trajectories of ten complexes are shown in Figures 6A,B. The ideal range for the stable complex in terms of RMSD is 2–3 Å. It can be observed that all the complexes showed a stable RMSD value i.e., less than ∼2.5 Å as compare to the apo TTK which showed higher deviation in confirmation than complexes. All the complexes equilibrated at ∼ 2 ns and then got stability till the end of the simulation. Some complexes showed higher stability than others i.e., TTK-NDC4 and TTK-NDC5 with a RMSD values less than ∼1.5 Å. Additionally, the behavior of apo protein was also tested which showed that the protein complexes were more stable than apo protein.

**FIGURE 7:**
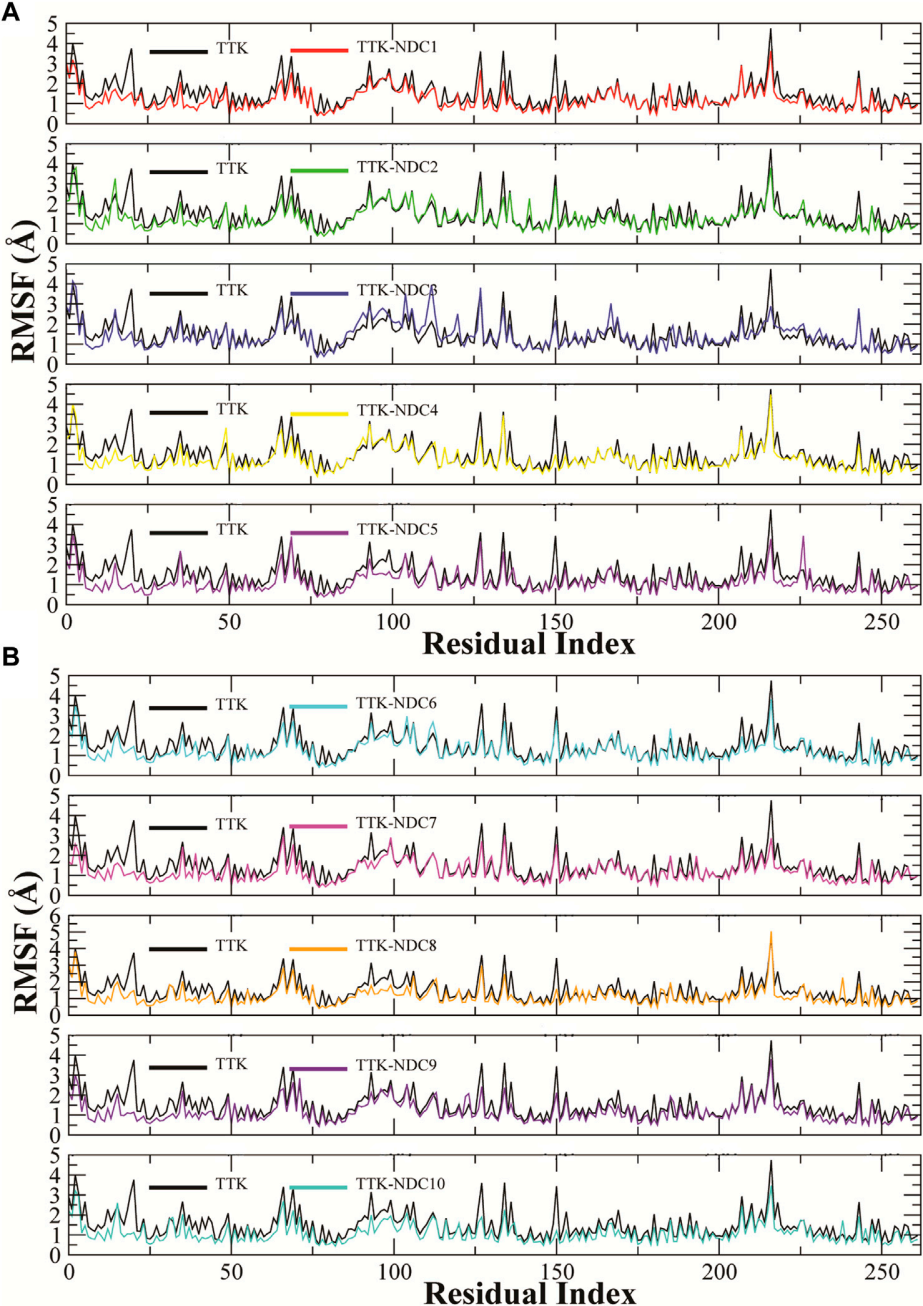
**(A,B).** Root Mean Square Fluctuations of amino acid residues of TTK protein and its complexes to compare the flexibility of protein structures.

The fluctuations in the amino acid residues were calculated by Root Mean Square Fluctuation (RMSF). The residues with high RMSF values showed higher flexibility, or the residues that form loop regions showed higher RMSF values. Similarly, the residues with lower RMSF values remained rigid during the simulation. Figures 7A,B describes the RMSF plots of all complexes. The C and N terminals showed highest RMSF values while the loop regions also showed relatively higher values than the rigid residues. All the complexes showed almost the same trend in RMSF values, with two regions having major fluctuations except for TTK-NDC3 and TTK-NDC6 complex. The major fluctuations were observed in the regions 85 to 115 and 210 to 225 residues. These correspond to the loop regions in the TKK protein while the other residues remained rigid having only a minor fluctuation in RMSF values.

**FIGURE 8:**
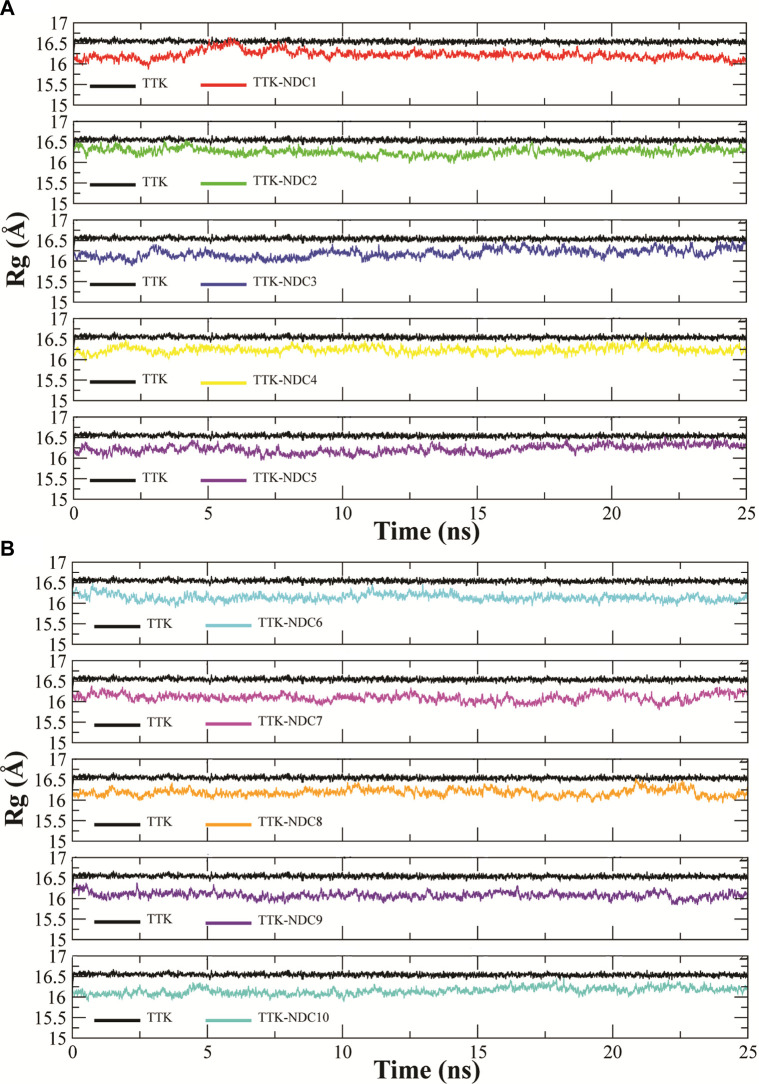
**(A,B)**. Radius of gyration of Cα atoms of TTK protein with bound compounds to analyze the relative compactness of protein complexes.

Rg (radius of gyration) is used to show the change in the structure compactness of subjected protein during simulations. The compactness shows that bound small molecules did not induce any conformational in the protein over the simulation time period (Seeliger and de Groot, 2010). Rg analysis represents how the secondary structures are compactly packed in 3D structure of proteins. The Rg plots of all complexes and apo TTK are shown in Figures 8A,B. TTK-NDC1 (black) had shown highest Rg during ∼5–8 ns, with Rg value reaching16.6 Å, while TTK-NDC2 (red) had shown the second highest value of ∼16.6 Å during first 5 ns simulations. The remaining complexes showed the stable Rg values throughout the simulation period. The stable Rg values indicated that the protein remained compact, and less unfolding was observed in all protein-ligand complexes as compare to the apo protein which showed higher Rg values than complexes throughout the simulation period (Grutsch et al., 2014).

**FIGURE 9:**
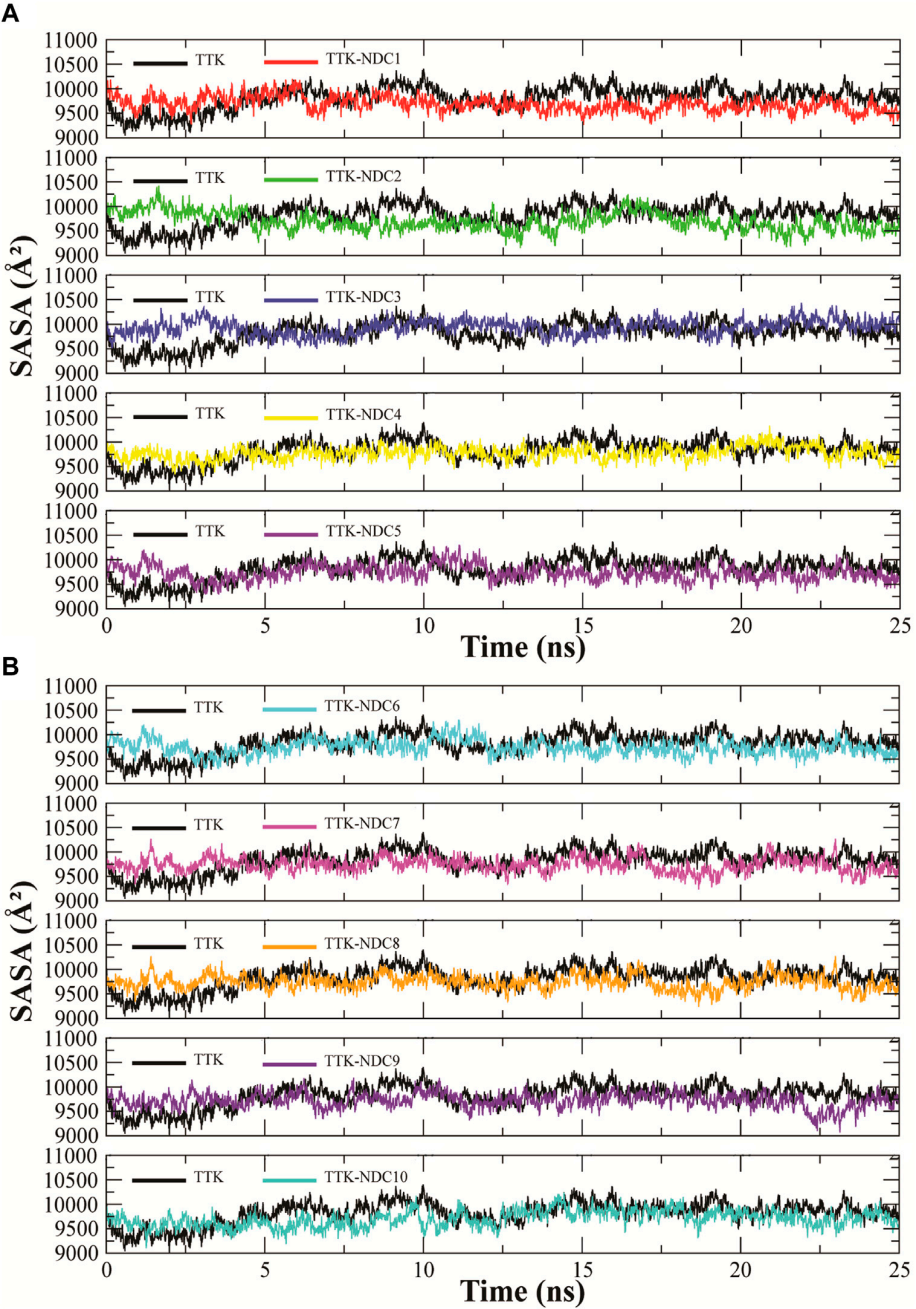
**(A,B)**. Solvent accessible surface area (SASA) calculation of TTK protein and its complexes throughout the simulation to find the exposed surface of protein to solvent during simulation.

SASA (solvent accessible surface area) is the surface area of a biomolecule that is accessible to a solvent. It determines how much an amino acid is exposed to its environment. Lower SASA values represent compact structure of protein while high values represent unfolded structures. SASA values of all TTK protein and its complexes were analyzed to predict the changes in the structure of the protein Figures 9A,B. The Figure shows that the proteins with ligands NDC2, NDC3 and NDC5 have higher SASA values while proteins with rest of the ligands have lowest SASA values. These results indicate that ligand binding can affect the protein’s tertiary structure. Increased values of SASA represent distortion in the structures.

**FIGURE 10:**
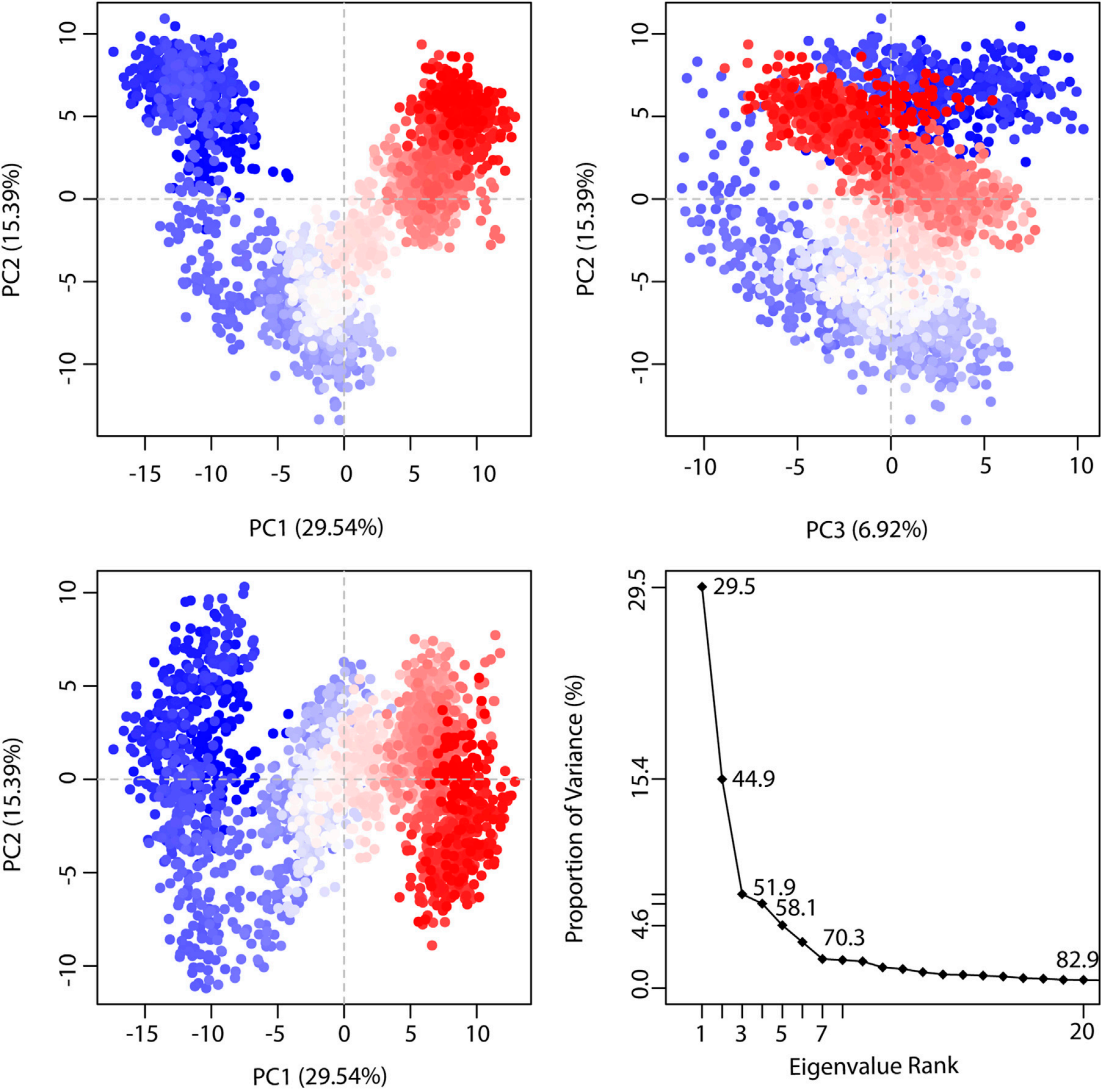
The representation of proportion of variance % (TTK-NDC1) against eigenvalue calculated by Principal Component Analysis. Three PCs are showing the fluctuating regions. The fluctuations in PC1, PC2, and PC3 are 29.54%, 15.39% and 6.92% respectively. The overall fluctuations are 51.85%.

PCA (Principal Component Analysis) characterizes the dynamic behavior of proteins (David and Jacobs, 2014). It helps to identify collective motions of the trajectories during MD simulations. In the graph of TTK-NDC1 (Figure 10), eigenvalues of the proteins were plotted against the corresponding eigenvector index for the first twenty modes of motion. The eigenvalues represent eigenvector fluctuations in hyperspace. In simulations overall movement of the proteins is controlled by eigenvectors with higher eigenvalues. In our systems, the first five eigenvectors exhibited dominant movements with a higher eigenvalue (29.5–70.3%), whereas the remaining eigenvectors had low eigenvalues. The plotted first three PC1, PC2 and PC3 covered the more than 50% of total variations. The Figure 10 plots shows that PC1 clusters possessed highest variability of 29.54%, PC2 depicted the variability of 15.39%, while PC3 exhibited minimal variability which is 6.92%. Minimal variability suggests that PC3 has the most stabilized protein ligand binding and occupies less region in phase space; hence its structure is compact as compare to PC1 and PC2. Through simple clustering in PC subspace, the PCA analysis revealed conformational changes in all clusters, blue regions showed most significant movement, white regions show intermediate movement while red regions show that there is less movement of flexibility. The PCA plots of remaining complexes are given in Supplementary Figures S1A–S1I.

**FIGURE 11:**
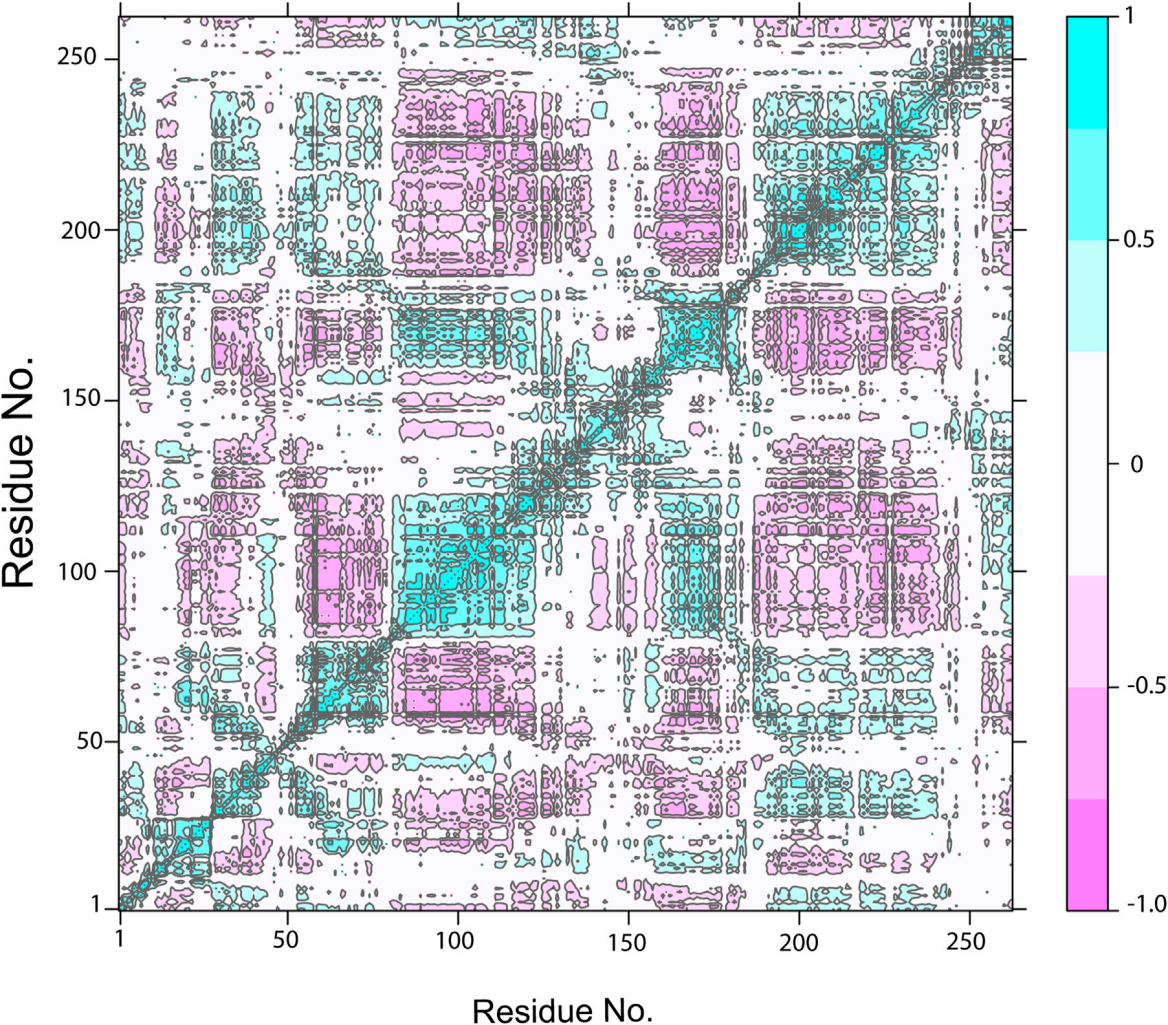
The Dynamic Cross-Correlation map of TTK-NDC1 complex. The positive and negative correlation among the residues is shown by cyan and purple color, respectively.

The cross-correlation map showed the pairwise correlation of NDC1 with the TTK protein by the value of pairwise cross-correlation coefficient (Figure 11). The correlated residues are more than 0.8 and are shown in cyan color, while the anti-correlated residues (<-0.4) are indicated with magenta color. The high percentage of pairwise correlated residues indicated the stable binding of the ligand with the TKK protein. The cross-correlation maps of other complexes are given in Supplementary Figures S2A–S2I.

### The binding free energy estimation

The MM/PBSA is a significant method to estimate the binding free energy of protein-ligand complexes. The ΔGbind values for all complexes were estimated using this method. The ΔG is the outcome of tcontribu-tion of various protein-ligand interactions such as van der Waals energy (ΔEvdW), electrostatic energy (ΔEele) and EPB (electrostatic contribution to solvation free energy by Poisson-Boltzmann) energy. The ΔEvdW of NDC9 and NDC10 complexes are found to be -78.76 kcal/mol and-72.07 kcal/mol respectively and contributing more in binding affinities as compared to other designed compounds. Whereas NDC8 complex having −29.34 kcal/mol showing its limited contribution whereas in remaining complexes it contributed more. In case of ΔEele contribution, the energy component is −5.85 kcal/mol in NDC1, 0.39 kcal/mol in NDC2, −4.09 kcal/mol in NDC3, −2.38 kcal/mol in NDC4, −8.55 kcal/mol in NDC5, −2.05 kcal/mol in NDC6, −2.57 kcal/mole in NDC7, −19.18 kcal/mol in NDC8, −5.04 kcal/mol in NDC9, and −5.22 kcal/mol in NDC10. ΔEele energy contribution of NDC8 complex is highest among all other complexes. Moreover, the PB contribution of all complexes are showing that NDC8 has higher PB value than other complexes. The calculated binding free energies of all the complexes are shown in Table 3. The binding free energies ΔGbind of NDC1 (−19.20 kcal/mol), NDC7(−18.71 kcal/mol) and NDC9 (−20.72 kcal/mol) are quite better than other complexes. The differences in the binding energies are due to the difference in the contribution of electrostatic, polar, and non-polar energies in the protein-ligand complexes.

**TABLE 3:** Components of the binding free energies of TTK and designed compounds complexes.

Complexes	ΔE_vdW_	ΔE_ele_	EPB	ΔG_NP_	ΔG_DIS_	ΔG_gas_	ΔG_solv_	ΔG_bind_
TTK-NDC1	−59.30 ± 0.34	−5.85± 0.30	18.54 ± 0.38	−28.92± 0.05	56.32 ± 0.20	−65.16 ± 0.46	45.95 ± 0.54	−19.20 ± 0.81
TTK-NDC2	−59.19 ± 0.30	0.39 ± 0.21	16.74 ± 0.33	−30.64± 0.13	61.17 ± 0.17	−58.84 ± 0.38	47.27 ± 0.40	−11.57 ± 0.43
TTK-NDC3	−54.15 ±0.20	−4.09 ± 0.23	18.27 ± 0.24	−27.20 ± 0.04	53.56 ± 0.09	−58.25 ± 0.31	44.63 ± 0.29	−13.61 ± 0.42
TTK-NDC4	−58.18 ± 0.20	−2.38 ± 0.18	16.47 ± 0.27	−28.00 ± 0.03	56.23 ± 0.08	−60.56 ± 0.24	44.70 ± 0.34	−15.86 ± 0.46
TTK-NDC5	−60.40 ± 0.25	−8.55 ± 0.23	30.06 ± 0.42	−28.38 ± 0.04	57.43 ± 0.09	−68.95 ± 0.32	59.10 ± 0.46	−9.85 ± 0.53
TTK-NDC6	−64.34 ± 0.33	−2.05 ± 0.17	27.69 ± 0.45	−33.11 ± 0.06	62.10 ± 0.11	−66.41 ± 0.34	56.68 ± 0.50	−9.73 ± 0.55
TTK-NDC7	−56.79 ± 0.26	−2.57 ± 0.20	14.23 ± 0.24	−27.97 ± 0.04	54.40 ± 0.10	−59.36 ± 0.30	40.65 ± 0.33	−18.71 ± 0.38
TTK-NDC8	−29.34 ± 0.21	−19.18 ± 0.45	48.81 ± 0.51	−39.58 ± 0.09	70.59 ± 0.12	−78.53 ± 0.51	79.82 ± 0.56	1.28 ± 0.40
TTK-NDC9	−78.76 ± 0.28	−5.04 ± 0.26	26.06 ± 0.28	−38.91 ± 0.08	75.29 ± 0.11	−83.80 ± 0.42	63.07 ± 0.35	−20.72 ± 0.55
TTK-NDC10	−72.07 ± 0.34	−5.22 ± 0.18	27.94 ± 0.37	−36.57 ± 0.12	73.83 ± 0.20	−77.30 ± 0.39	65.21 ± 0.47	−12.08 ± 0.41

### Calculations of physicochemical properties

QikProp software was used to estimate the physicochemical parameters (Table 4). With almost one rule violation, the majority of newly created molecules followed the Lipinski’s rule. The predicted octanol/water partition coefficient ‘QPlogPo/w’ values range (10.346–16.905), HERG K+ channels “QPlogHERG” blocking IC50 values range (−7.032 to −5.518), caco-2 cell permeability “QPPCaco’” values range (66.578–2019.982), brain/blood partition coefficient “QPlogBB” values range (−1.9 to −0.427), and human serum albumin binding “QPkhsa” values range (−0.155–0.947) are within the acceptable ranges for 95 percent oral drugs (Kumar et al., 2016). Within the recommended ranges, physicochemical qualities such as ‘QPlogPo/w and QPlogHERG’ showed smooth diffusion of drug and protection against unexpected cardiac arrest (Kumar et al., 2016).

**TABLE 4:** Predicted physicochemical properties of the newly designed molecules.

Compounds	MW	HBD	HBA	QPlogPo/w	QPlogHERG	QPCaco	QPlogBB	QPlogKhsa
NDC1	320.35	3	5	13.329	−6.193	620.519	−0.945	0.086
NDC2	349.391	2	4	10.346	−6.155	2019.982	−0.427	0.545
NDC3	307.311	5	4	15.776	−5.889	66.578	−1.9	−0.152
NDC4	305.338	4	5	16.231	−6.195	145.681	−1.599	−0.155
NDC5	324.385	5	4	15.72	−5.619	366.227	−1.176	−0.038
NDC6	388.351	4	4	14.646	−5.949	291.298	−1.059	0.217
NDC7	307.354	5	3	14.015	−5.518	189.325	−1.36	0.091
NDC8	504.73	8	8	−0.743	−7.367	1.276	−1.203	−0.007
NDC9	470.573	4	6	15.944	−5.648	401.322	−1.072	0.759
NDC10	482.584	4	6	16.905	−7.032	244.531	−1.612	0.947

“QPlogPo/w recommended range = “−2.0 to 6.5,” QPlogHERG recommended range = “<-5,” QPCaco2 recommended range “<25 poor,” “> 500 great,” QPlogBB recommended range = “−3.0 to 1.2,” QPlogKhsa recommended range = “−1.5 to 1.5.”

## Conclusion

TTK is an important mitotic kinase whose loss of function results in chromosomal segregation defects that can lead to aneuploidy and cell death, making it an attractive drug target for cancer. Using different partial charges and alignment methods, structure-based 3D-QSAR models on MMFF94 charges yielded best CoMFA and CoMSIA models. Using these predictive models and contour maps information ten new compounds were designed and their biological activities were predicted. The newly designed compounds showed better predicted activities than their parent compounds, demonstrating that structure-based approaches using MMFF94 charges can be used to design better active TTK inhibitors. Further MD simulations described the stability of protein-ligand complexes. Similarly computational binding free energy calculations suggest that newly designed compounds can bind to TTK protein with better binding affinity than reported compounds.

## Data Availability

The original contributions presented in the study are included in the article/Supplementary Material, further inquiries can be directed to the corresponding authors.

## References

[B1] Acun B. Hardy D. J. Kale L. V. Li K. Phillips J. C. Stone J. E. (2018). Scalable molecular dynamics with NAMD on the Summit system. IBM J. Res. Dev. 62 (4), 1–9. 10.1147/jrd.2018.2888986 PMC7059615 32154805

[B2] Balasubramanian P. K. Balupuri A. Cho S. J. (2014). A CoMFA study of phenoxypyridine-based JNK3 inhibitors using various partial charge schemes. J. Chosun Nat. Sci. 7, 45–49. 10.13160/ricns.2014.7.1.45

[B3] Bhachoo J. Beuming T. J. M. P.-P. I. (2017). Investigating protein–peptide interactions using the Schrödinger computational suite. Methods Mol. Biol. 1561, 235–254. 10.1007/978-1-4939-6798-8_14 28236242

[B4] Brough R. Frankum J. R. Sims D. Mackay A. Mendes-Pereira A. M. Bajrami I. (2011). Functional viability profiles of breast cancer. Cancer Discov. 1, 260–273. 10.1158/2159-8290.CD-11-0107 21984977 PMC3188379

[B5] Bursavich M. G. Dastrup D. Shenderovich M. Yager K. M. Cimbora D. M. Williams B. (2013). Novel Mps1 kinase inhibitors: From purine to pyrrolopyrimidine and quinazoline leads. Bioorg. Med. Chem. Lett. 23, 6829–6833. 10.1016/j.bmcl.2013.10.008 24183538

[B6] Case D. A. Aktulga H. M. Belfon K. Ben-Shalom I. Brozell S. R. Cerutti D. (2021). Amber 2021: Reference manual. Covers Amber20 AmberTools21. 10.13140/RG.2.2.15902.66881

[B7] Chen Y. Yu W. Jiang C.-C. Zheng J.-G. J. M. (2018). Insights into resistance mechanisms of inhibitors to Mps1 C604Y mutation via a comprehensive molecular modeling study. Molecules 23, 1488. 10.3390/molecules23061488 PMC6100145 29925769

[B8] Daniel J. Coulter J. Woo J.-H. Wilsbach K. Gabrielson E. J. P. O. T. N. A. O. S. (2011). High levels of the Mps1 checkpoint protein are protective of aneuploidy in breast cancer cells. Proc. Natl. Acad. Sci. U. S. A. 108, 5384–5389. 10.1073/pnas.1007645108 21402910 PMC3069188

[B9] David C. C. Jacobs D. J. (2014). Principal component analysis: A method for determining the essential dynamics of proteins. Methods Mol. Biol. 1084, 193–226. 10.1007/978-1-62703-658-0_11 24061923 PMC4676806

[B10] Duan Y. Wu C. Chowdhury S. Lee M. C. Xiong G. Zhang W. (2003). A point‐charge force field for molecular mechanics simulations of proteins based on condensed‐phase quantum mechanical calculations. J. Comput. Chem. 24, 1999–2012. 10.1002/jcc.10349 14531054

[B11] Fisk H. A. Winey M. (2001). The mouse Mps1p-like kinase regulates centrosome duplication. Cell 106, 95–104. 10.1016/s0092-8674(01)00411-1 11461705

[B12] Ghosh S. Keretsu S. Cho S. J. (2021). Designing of the N-ethyl-4-(pyridin-4-yl) benzamide based potent ROCK1 inhibitors using docking, molecular dynamics, and 3D-QSAR. PeerJ 9, e11951. 10.7717/peerj.11951 34434664 PMC8359802

[B13] Grutsch S. Fuchs J. E. Freier R. Kofler S. Bibi M. Asam C. (2014). Ligand binding modulates the structural dynamics and compactness of the major birch pollen allergen. Biophys. J. 107, 2972–2981. 10.1016/j.bpj.2014.10.062 25517162 PMC4269767

[B14] Hu R. Barbault F. Delamar M. Zhang R. (2009). Receptor-and ligand-based 3D-QSAR study for a series of non-nucleoside HIV-1 reverse transcriptase inhibitors. Bioorg. Med. Chem. 17, 2400–2409. 10.1016/j.bmc.2009.02.003 19250835

[B15] Huang M. Huang Y. Guo J. Yu L. Chang Y. Wang X. (2021). Pyrido [2, 3-d] pyrimidin-7 (8H)-ones as new selective orally bioavailable Threonine Tyrosine Kinase (TTK) inhibitors. Eur. J. Med. Chem. 211, 113023. 10.1016/j.ejmech.2020.113023 33248853

[B16] Koç E. Üngördü A. Candan F. J. S. C. (2021). Antioxidant activities of Alyssum virgatum plant and its main components. Struct. Chem. 33, 267–279. 10.1007/s11224-021-01856-1

[B17] Kumar A. Ito A. Hirohama M. Yoshida M. Zhang K. Y. (2016). Identification of new SUMO activating enzyme 1 inhibitors using virtual screening and scaffold hopping. Bioorg. Med. Chem. Lett. 26, 1218–1223. 10.1016/j.bmcl.2016.01.030 26810265

[B18] Kusakabe K.-I. Ide N. Daigo Y. Itoh T. Higashino K. Okano Y. (2012). Diaminopyridine-based potent and selective Mps1 kinase inhibitors binding to an unusual flipped-peptide conformation. ACS Med. Chem. Lett. 3, 560–564. 10.1021/ml3000879 24900510 PMC4025729

[B19] Laufer R. Ng G. Liu Y. Patel N. K. B. Edwards L. G. Lang Y. (2014). Discovery of inhibitors of the mitotic kinase TTK based on N-(3-(3-sulfamoylphenyl)-1H-indazol-5-yl)-acetamides and carboxamides. Bioorg. Med. Chem. 22, 4968–4997. 10.1016/j.bmc.2014.06.027 25043312

[B20] Li S. Fan J. Peng C. Chang Y. Guo L. Hou J. (2017). New molecular insights into the tyrosyl-tRNA synthase inhibitors: CoMFA, CoMSIA analyses and molecular docking studies. Sci. Rep. 7, 1–13. 10.1038/s41598-017-10618-1 28912450 PMC5599502

[B21] Lindberg M. F. Meijer L. (2021). Dual-specificity, tyrosine phosphorylation-regulated kinases (DYRKs) and cdc2-like kinases (CLKs) in human disease, an overview. Int. J. Mol. Sci. 22, 6047. 10.3390/ijms22116047 34205123 PMC8199962

[B22] Liu X. Liao W. Yuan Q. Ou Y. Huang J. (2015a). TTK activates Akt and promotes proliferation and migration of hepatocellular carcinoma cells. Oncotarget 6, 34309–34320. 10.18632/oncotarget.5295 26418879 PMC4741454

[B23] Liu X. Winey M. (2012). The MPS1 family of protein kinases. Annu. Rev. Biochem. 81, 561–585. 10.1146/annurev-biochem-061611-090435 22482908 PMC4026297

[B24] Liu Y. Lang Y. Patel N. K. Ng G. Laufer R. Li S.-W. (2015b). The discovery of orally bioavailable tyrosine threonine kinase (TTK) inhibitors: 3-(4-(heterocyclyl) phenyl)-1 H-indazole-5-carboxamides as anticancer agents. J. Med. Chem. 58, 3366–3392. 10.1021/jm501740a 25763473

[B25] Lorca M. Valdes Y. Chung H. Pessoa-Mahana C. D. Mella J. 2018. 3D-QSAR on a series of pyrimidinyl-piperazine-carboxamides based Fatty Acid Amide Hydrolase (FAAH) inhibitors as a useful tool to obtain novel endocannabinoid enhancers, Preprints 2018. 10.3390/ijms20102510 PMC6566251 31117309

[B26] Lu N. Ren L. J. B. (2021). TTK (threonine tyrosine kinase) regulates the malignant behaviors of cancer cells and is regulated by microRNA-582-5p in ovarian cancer. Bioengineered 12, 5759–5768. 10.1080/21655979.2021.1968778 34516342 PMC8806697

[B27] Maia A. R. De Man J. Boon U. Janssen A. Song J.-Y. Omerzu M. (2015). Inhibition of the spindle assembly checkpoint kinase TTK enhances the efficacy of docetaxel in a triple-negative breast cancer model. Ann. Oncol. 26, 2180–2192. 10.1093/annonc/mdv293 26153498

[B28] Muddassar M. Jang J. W. Hong S. K. Cho Y. S. Kim E. E. Keum K. C. (2010). Identification of novel antitubercular compounds through hybrid virtual screening approach. Bioorg. Med. Chem. 18, 6914–6921. 10.1016/j.bmc.2010.07.010 20727773

[B29] Muddassar M. Pasha F. Neaz M. Saleem Y. Cho S. (2009). Elucidation of binding mode and three dimensional quantitative structure–activity relationship studies of a novel series of protein kinase B/Akt inhibitors. J. Mol. Model. 15, 183–192. 10.1007/s00894-008-0416-7 19043747

[B30] Naud S. Westwood I. M. Faisal A. Sheldrake P. Bavetsias V. Atrash B. (2013). Structure-based design of orally bioavailable 1H-Pyrrolo[3, 2-c]pyridine inhibitors of mitotic kinase monopolar spindle 1 (MPS1). J. Med. Chem. 56, 10045–10065. 10.1021/jm401395s 24256217 PMC3873811

[B31] Puzyn T. Mostrag-Szlichtyng A. Gajewicz A. Skrzyński M. Worth A. P. (2011). Investigating the influence of data splitting on the predictive ability of QSAR/QSPR models. Struct. Chem. 22, 795–804. 10.1007/s11224-011-9757-4

[B32] Roe D. R. Cheatham T. E. J. J. O. C. T. III (2013). PTRAJ and CPPTRAJ: Software for processing and analysis of molecular dynamics trajectory data. J. Chem. Theory Comput. 9, 3084–3095. 10.1021/ct400341p 26583988

[B33] Sainy J. Sharma R. (2015). QSAR analysis of thiolactone derivatives using HQSAR, CoMFA and CoMSIA. SAR QSAR Environ. Res. 26, 873–892. 10.1080/1062936x.2015.1095238 26524489

[B34] Schmidt M. Budirahardja Y. Klompmaker R. Medema R. H. J. E. R. (2005). Ablation of the spindle assembly checkpoint by a compound targeting Mps1. EMBO Rep. 6, 866–872. 10.1038/sj.embor.7400483 16113653 PMC1369161

[B35] Seeliger D. De Groot B. L. (2010). Conformational transitions upon ligand binding: Holo-structure prediction from apo conformations. PLoS Comput. Biol. 6, e1000634. 10.1371/journal.pcbi.1000634 20066034 PMC2796265

[B36] Shiri F. Rakhshani-Morad S. Samzadeh-Kermani A. Karimi P. (2016). Computer-aided molecular design of some indolinone derivatives of PLK4 inhibitors as novel anti-proliferative agents. Med. Chem. Res. 25, 2643–2665. 10.1007/s00044-016-1638-3

[B37] Stucke V. M. Silljé H. H. Arnaud L. Nigg E. A. (2002). Human Mps1 kinase is required for the spindle assembly checkpoint but not for centrosome duplication. EMBO J. 21, 1723–1732. 10.1093/emboj/21.7.1723 11927556 PMC125937

[B38] Sugimoto Y. Sawant D. B. Fisk H. A. Mao L. Li C. Chettiar S. (2017b). Novel pyrrolopyrimidines as Mps1/TTK kinase inhibitors for breast cancer. Bioorg. Med. Chem. 25, 2156–2166. 10.1016/j.bmc.2017.02.030 28259529

[B39] Sugimoto Y. Sawant D. B. Fisk H. A. Mao L. Li C. Chettiar S. (2017a). Novel pyrrolopyrimidines as Mps1/TTK kinase inhibitors for breast cancer. Bioorg. Med. Chem. 25, 2156–2166. 10.1016/j.bmc.2017.02.030 28259529

[B40] Sun H. Li Y. Shen M. Tian S. Xu L. Pan P. (2014). Assessing the performance of MM/PBSA and MM/GBSA methods. 5. Improved docking performance using high solute dielectric constant MM/GBSA and MM/PBSA rescoring. Phys. Chem. Chem. Phys. 16, 22035–22045. 10.1039/c4cp03179b 25205360

[B41] Tahir A. Alharthy R. D. Naseem S. Mahmood N. Ahmed M. Shahzad K. (2018). Investigations of structural requirements for brd4 inhibitors through ligand-and structure-based 3D QSAR approaches. Molecules 23, 1527. 10.3390/molecules23071527 PMC6099705 29941841

[B42] Vaidya A. Jain A. K. Kumar B. P. Sastry G. Kashaw S. K. Agrawal R. K. (2017). CoMFA, CoMSIA, kNN MFA and docking studies of 1, 2, 4-oxadiazole derivatives as potent caspase-3 activators. Arabian J. Chem. 10, S3936–S3946. 10.1016/j.arabjc.2014.05.034

[B43] Wang F. Yang W. Shi Y. Le G. (2015). Structural analysis of selective agonists of thyroid hormone receptor β using 3D-QSAR and molecular docking. J. Taiwan Inst. Chem. Eng. 49, 1–18. 10.1016/j.jtice.2014.11.009

[B44] Wang W. Yang Y. Gao Y. Xu Q. Wang F. Zhu S. (2009). Structural and mechanistic insights into Mps1 kinase activation. J. Cell. Mol. Med. 13, 1679–1694. 10.1111/j.1582-4934.2008.00605.x 19120698 PMC2829362

[B45] Wei J.-H. Chou Y.-F. Ou Y.-H. Yeh Y.-H. Tyan S.-W. Sun T.-P. (2005). TTK/hMps1 participates in the regulation of DNA damage checkpoint response by phosphorylating CHK2 on threonine 68. J. Biol. Chem. 280, 7748–7757. 10.1074/jbc.m410152200 15618221

[B46] Wengner A. M. Siemeister G. Koppitz M. Schulze V. Kosemund D. Klar U. (2016). Novel Mps1 kinase inhibitors with potent antitumor activity. Mol. cancer Ther. 15, 583–592. 10.1158/1535-7163.mct-15-0500 26832791

[B47] Xing C. Zhou X. Chen C. Sun W. Zheng Q. Liang D. (2021). Studies of interaction mechanism between pyrido [3, 4-d] pyrimidine inhibitors and Mps1. Molecules 26, 5075. 10.3390/molecules26165075 34443663 PMC8401005

