# Supplementary material for: Combined 3D-QSAR, molecular docking and dynamics simulations studies to model and design TTK inhibitors
Source: Front Chem. 2022 Nov 2;10:1003816. doi: 10.3389/fchem.2022.1003816 (PMC9666879; doi:10.3389/fchem.2022.1003816)
Supplement: Supplementary file 1 [file DataSheet1.pdf]

# *Supplementary Material*

## Combined 3D-QSAR, Molecular docking and dynamics simulations studies to model and design TTK inhibitors

**Noureen Ashraf †[1], Numan Yousaf †[1], Asnuzilawati Asari*[2], Matloob Ahmad[3], Mahmood Ahmed[4], Amir Faisal[5], Muhammad Saleem[6], Muhammad Muddassar*[1]**

[1]Department of Biosciences, COMSATS University Islamabad, Park Road, Islamabad, Pakistan.

[2]Faculty of Science and Marine Environment, University Malaysia Terengganu, 21030 Kuala Nerus, Terengganu, Malaysia.

[3]Department of Chemistry, Government College University, Faisalabad 38000, Pakistan.

[4]Department of Chemistry, Division of Science and Technology, University of Education, Lahore-Pakistan

[5]Department of Biology, Syed Babar Ali School of Science and Engineering, Lahore University of Management Sciences, Lahore, Pakistan.

[6]School of Biological Sciences, University of the Punjab, Lahore, Pakistan.

* Correspondence: mmuddassar@comsats.edu.pk; Tel: (+92-331-6042666) and asnu@umt.edu.my  Tel: +60 14-216 2751

**Table S1|** Structures and Biological Activities of TTK Inhibitors

| Sr. No. | Compound | Structure | pIC 50 | Sr. No. | Compound | Structure | pIC 50 |
|---|---|---|---|---|---|---|---|
| **1** | 1 | 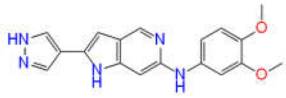 | 7.602 | **9** | **9** | 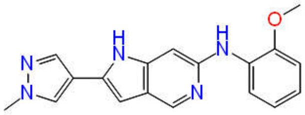 | 6.920 |
| **2** | 2* | 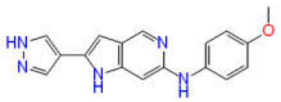 | 7.292 | **10** | **10** | 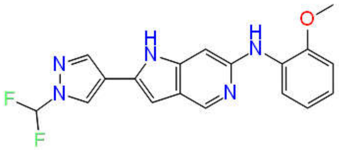 | 6.34 |
| **3** | 3* | 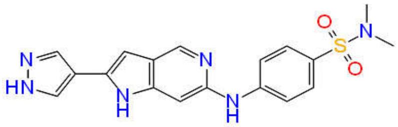 | **7.80** | **11** | **11** | 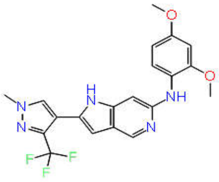 | 6.004 |
| **4** | **4** | 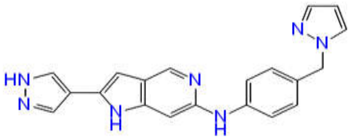 | 7.68 | **12** | **12** | 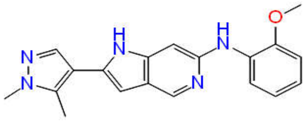 | 5.80 |
| **5** | **5** | 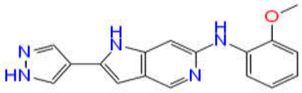 | 7.602 | **13** | **13** | 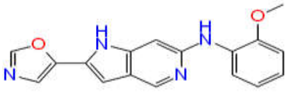 | 7.59 |
| **6** | **6** | 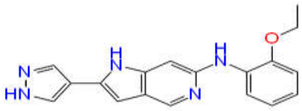 | 7.24 | **14** | **14** | 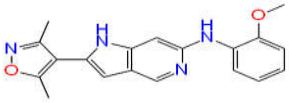 | 5.31 |
| **7** | **7** | 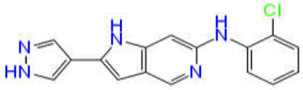 | 7.56 | **15** | **15** | 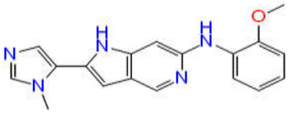 | 6.193 |
| **8** | **8** | 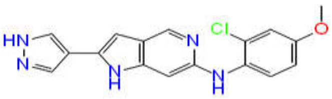 | 7.70 | **16** | **16*** | 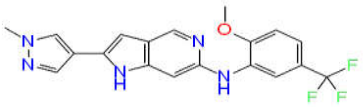 | 5.36 |

| Sr. No. | Compound | Structure | pIC50 | Sr. No. | Compound | Structure | pIC50 |
|---|---|---|---|---|---|---|---|
| **17** | 17 | 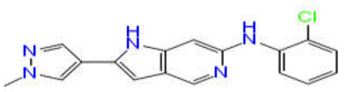 | 7.130 | **25** | **25*** | 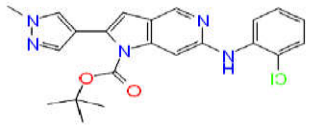 | 7.148 |
| **18** | 18* | 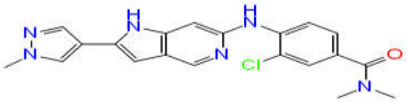 | 7.823 | **26** | **26** | 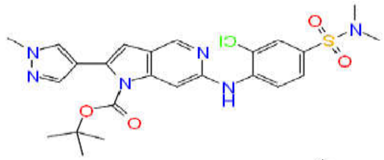 | 7.036 |
| **19** | 19 | 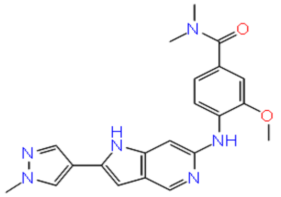 | 7.638 | **27** | **27*** | 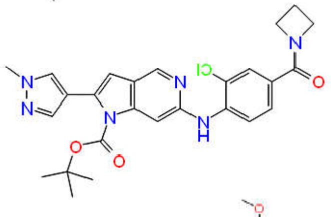 | 8.154 |
| **20** | **20** | 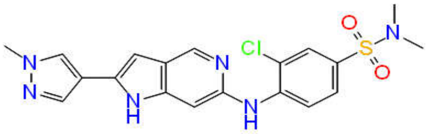 | 7.57 | **28** | **28** | 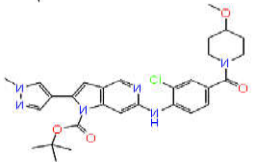 | 7.96 |
| **21** | **21*** | 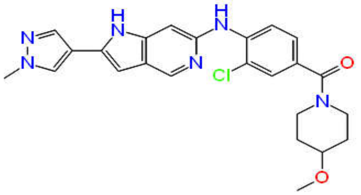 | 7.89 | **29** | **29** | 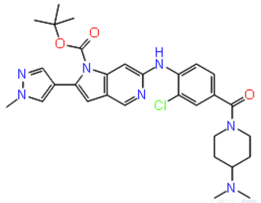 | 8.40 |
| **22** | **22*** | 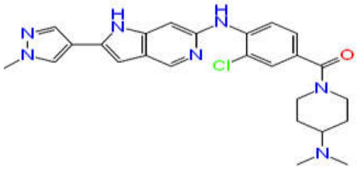 | 7.70 | **30** | **30** | 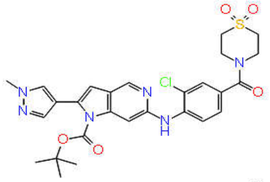 | 8.301 |
| **23** | **23*** | 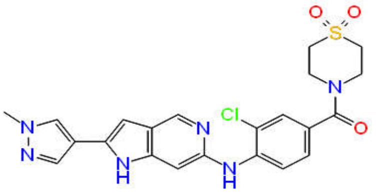 | 8.10 | **31** | **31** | 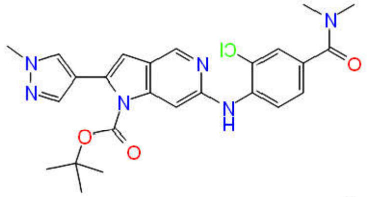 | 7.66 |
| **24** | **24*** | 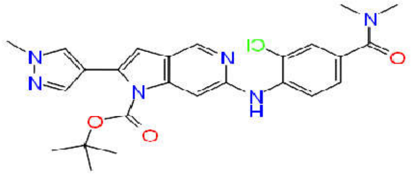 | 8.221 | **32** | **32** | 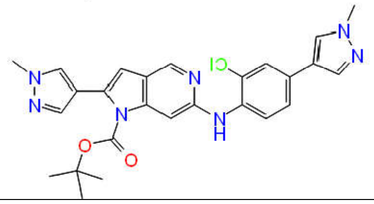 | 7.823 |

| Sr. No. | Compound | Structure | pIC 50 | Sr. No. | Compound | Structure | pIC 50 |
|---|---|---|---|---|---|---|---|
| **33** | 33 | 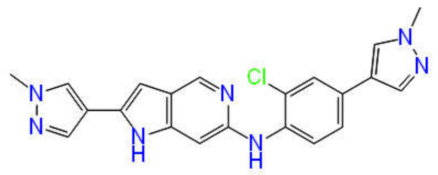 | 7.77 | **37** | **37** | 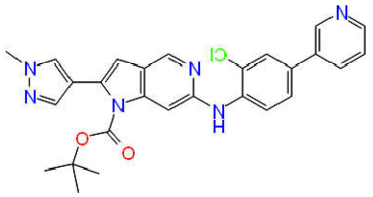 | 7.56 |
| **34** | 34 | 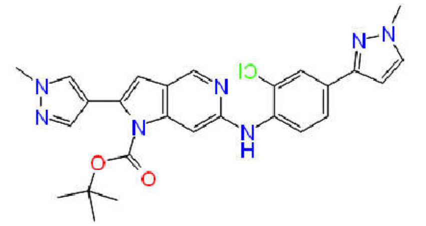 | 7.70 | **38** | **38*** | 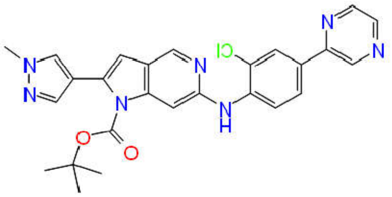 | 7.494 |
| **35** | 35 | 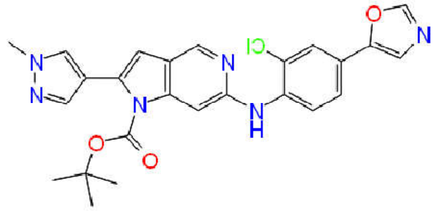 | 7.721 | **39** | **39** | 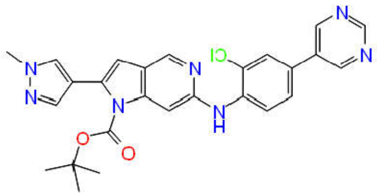 | 7.68 |
| **36** | **36** | 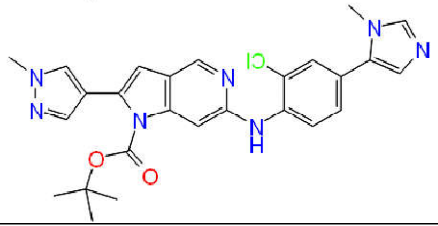 | 8.522 | | | | |

***Test compounds**

**Table S2|** Results of Ligand Based (Powell Method) 3D QSAR models with Different Charges

| | Ligand Based Model 39 Compounds | | | | | | | |
|---|---|---|---|---|---|---|---|---|
| | Gasteiger Huckel Charges (GH) | | Gasteiger Marsili Charges (GM) | | Pullman Charges (PM) | | Merck Molecular Force Field (MMFF94) | |
| **Parameters** | **CoMFA** | **CoMSIA** | **CoMFA** | **CoMSIA** | **CoMFA** | **CoMSIA** | **CoMFA** | **CoMSIA** |
| **N** | 3 | 4 | 2 | 4 | 3 | 4 | 2 | 4 |
| **$q^2$(LOO)** | 0.262 | 0.509 | 0.217 | 0.464 | 0.268 | 0.503 | 0.212 | 0.461 |
| **$r^2$(NoV)** | 0.818 | 0.944 | 0.630 | 0.929 | 0.811 | 0.914 | 0.658 | 0.909 |
| **SEE** | 0.351 | 0.199 | 0.492 | 0.225 | 0.359 | 0.247 | 0.472 | 0.254 |
| **F** | 36.064 | 97.835 | 21.280 | 75.086 | 34.319 | 61.147 | 24.101 | 57.524 |
| **Pred ($r^{2)}$** | 0.596 | 0.843 | 0.332 | 0.796 | 0.621 | 0.805 | 0.330 | 0.838 |
| **Steric (S)** | 0.658 | 0.136 | 0.911 | 0.137 | 0.685 | 0.147 | 0.771 | 0.136 |
| **Electrostatic(E)** | 0.342 | 0.243 | 0.089 | 0.169 | 0.315 | 0.211 | 0.229 | 0.206 |
| **Hydrophobic (H)** | ----- | 0.267 | ----- | 0.277 | ----- | 0.278 | ----- | 0.272 |
| **Donor (D)** | ----- | 0.172 | ----- | 0.185 | ----- | 0.178 | ----- | 0.176 |
| **Acceptor (A)** | ----- | 0.182 | ----- | 0.232 | ----- | 0.185 | ----- | 0.209 |
| **$r^2$bs** | 0.984 | 0.994 | 0.982 | 0.992 | 0.980 | 0.992 | 0.981 | 0.990 |
| **SDbs** | 0.011 | 0.003 | 0.012 | 0.005 | 0.011 | 0.005 | 0.016 | 0.006 |
| **$r^2$cv** | 0.328 | 0.496 | 0.241 | 0.432 | 0.271 | 0.487 | 0.226 | 0.433 |

optimal number of components = ONC; cross-validated correlation coefficient = q2; determination coefficient= r2; non-cross validated coefficient= r2 nov; standard error of estimate= SEE; Fischer's F-value=F; predictive r2= Pred-r2; r2 obtained after bootstrapping= r2 bs; and bootstrapping standard deviation= $SD_{bs}$.

**Table S3|** Results of Ligand Based (Conjugate Gradient Method) 3D QSAR models with Different Charges

| | Ligand Based Model 39 Compounds | | | | | | | |
|---|---|---|---|---|---|---|---|---|
| | **Gasteiger Huckel Charges (GH)** | | **Gasteiger Marsili Charges (GM)** | | **Pullman Charges (PM)** | | **Merck Molecular Force Field (MMFF94)** | |
| **Parameters** | **CoMFA** | **CoMSIA** | **CoMFA** | **CoMSIA** | **CoMFA** | **CoMSIA** | **CoMFA** | **CoMSIA** |
| **N** | 3 | 4 | 3 | 3 | 3 | 4 | 2 | 4 |
| **$q^2$(LOO)** | 0.240 | 0.496 | 0.220 | 0.454 | 0.244 | 0.497 | 0.191 | 0.459 |
| **$r^2$(NoV)** | 0.824 | 0.942 | 0.814 | 0.815 | 0.817 | 0.911 | 0.880 | 0.903 |
| **SEE** | 0.346 | 0.203 | 0.356 | 0.355 | 0.353 | 0.251 | 0.292 | 0.263 |
| **F** | 37.373 | 93.390 | 35.045 | 35.198 | 35.662 | 59.056 | 42.066 | 53.275 |
| **Pred ($r^{2)}$** | 0.599 | 0.857 | 0.588 | 0.717 | 0.618 | 0.818 | 0.769 | 0.845 |
| **Steric (S)** | 0.643 | 0.138 | 0.728 | 0.154 | 0.672 | 0.149 | 0.676 | 0.139 |
| **Electrostatic(E)** | 0.357 | 0.242 | 0.272 | 0.136 | 0.328 | 0.215 | 0.324 | 0.197 |
| **Hydrophobic (H)** | ----- | 0.267 | ----- | 0.280 | ----- | 0.277 | ----- | 0.278 |
| **Donor (D)** | ----- | 0.170 | ----- | 0.188 | ----- | 0.176 | ----- | 0.173 |
| **Acceptor (A)** | ----- | 0.184 | ----- | 0.242 | ----- | 0.183 | ----- | 0.213 |
| **$r^2$bs** | 0.982 | 0.992 | 0.987 | 0.990 | 0.987 | 0.992 | 0.923 | 0.935 |
| **SDbs** | 0.007 | 0.005 | 0.008 | 0.004 | 0.008 | 0.006 | 0.028 | 0.037 |

optimal number of components = ONC; cross-validated correlation coefficient = q2; determination coefficient= r2; non-cross validated coefficient= r2 nov; standard error of estimate= SEE; Fischer's F-value=F; predictive r2= Pred-r2; r2 obtained after bootstrapping= r2 bs; and bootstrapping standard deviation= $SD_{bs}$

**Table S4|** Docking scores of new designed compounds

| No. | Compounds | Structure | Glide gscore (kcal/mol) |
|---|---|---|---|
| 1 | NDC1 | 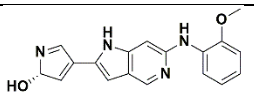 | -8.959 |
| 2 | NDC2 | 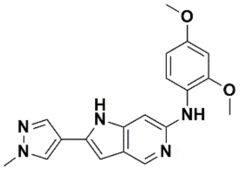 | -8.329 |
| 3 | NDC3 | 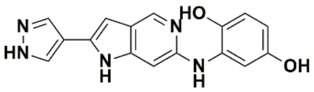 | -9.113 |
| 4 | NDC4 | 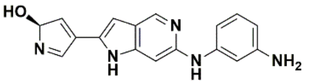 | -9.353 |
| 5 | NDC5 | 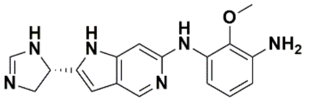 | -7.412 |
| 6 | NDC6 | 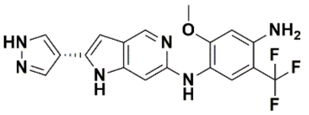 | -8.169 |
| 7 | NDC7 | 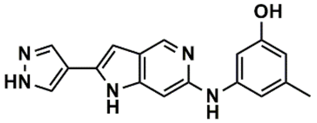 | -8.787 |
| 8 | NDC8 | 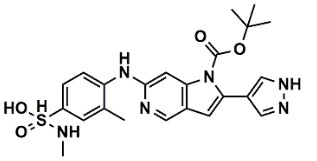 | -10.385 |
| 9 | NDC9 | 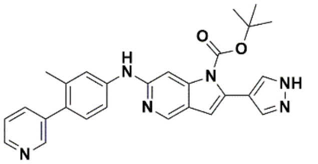 | -10.953 |
| 10 | NDC10 | 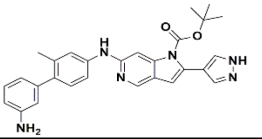 | -12.019 |

**Table S5|** Average RMSD values of all complexes

| Complex | Average RMSD |
|---|---|
| TTK-NDC1 | 1.57±0.15 |
| TTK-NDC2 | 1.72±0.19 |
| TTK-NDC3 | 1.82±0.27 |
| TTK-NDC4 | 1.45±0.16 |
| TTK-NDC5 | 1.30±0.15 |
| TTK-NDC6 | 1.78±0.28 |
| TTK-NDC7 | 1.66±0.26 |
| TTK-NDC8 | 1.51±0.29 |
| TTK-NDC9 | 1.41±0.21 |
| TTK-NDC10 | 1.57±0.16 |

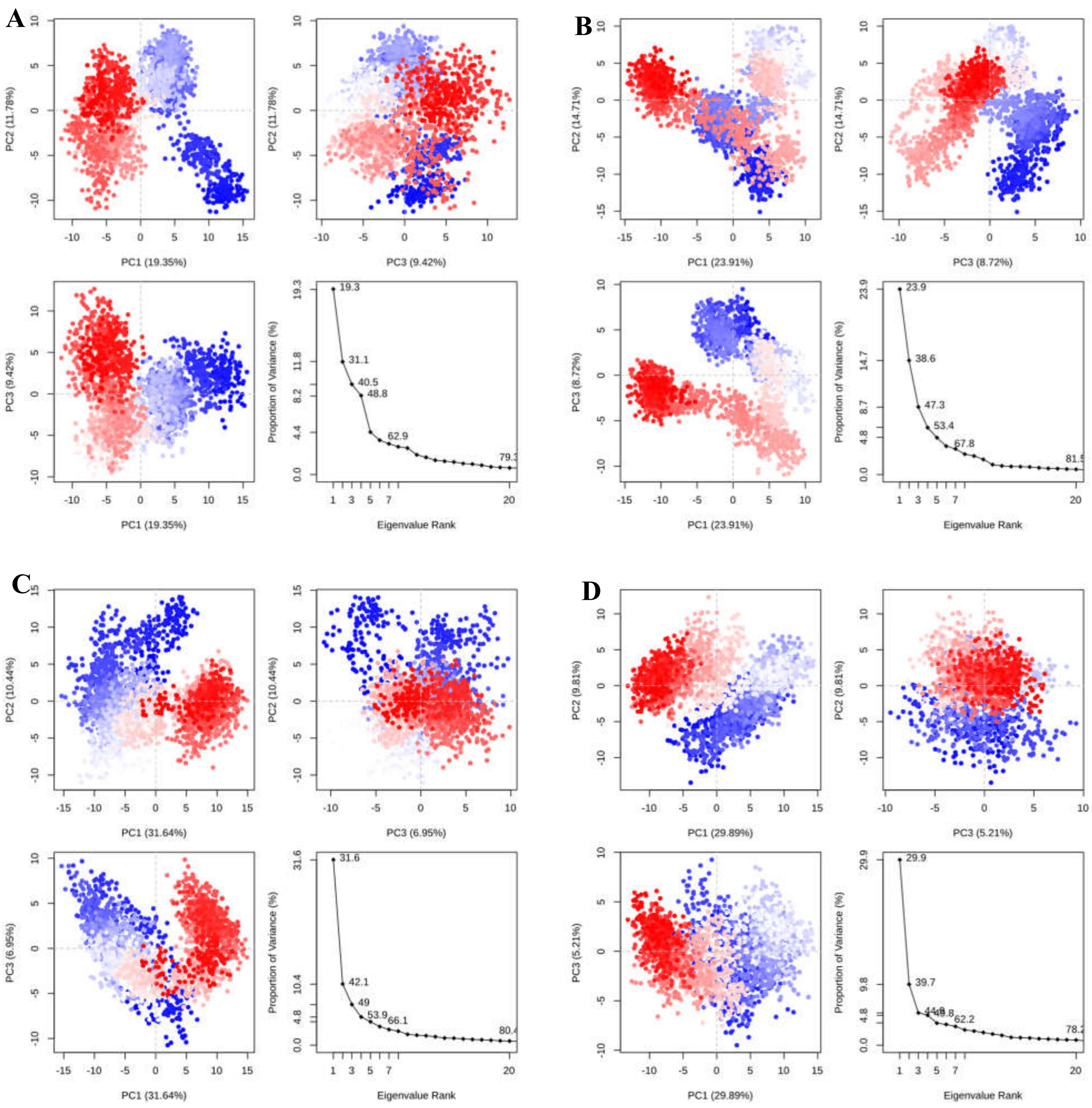

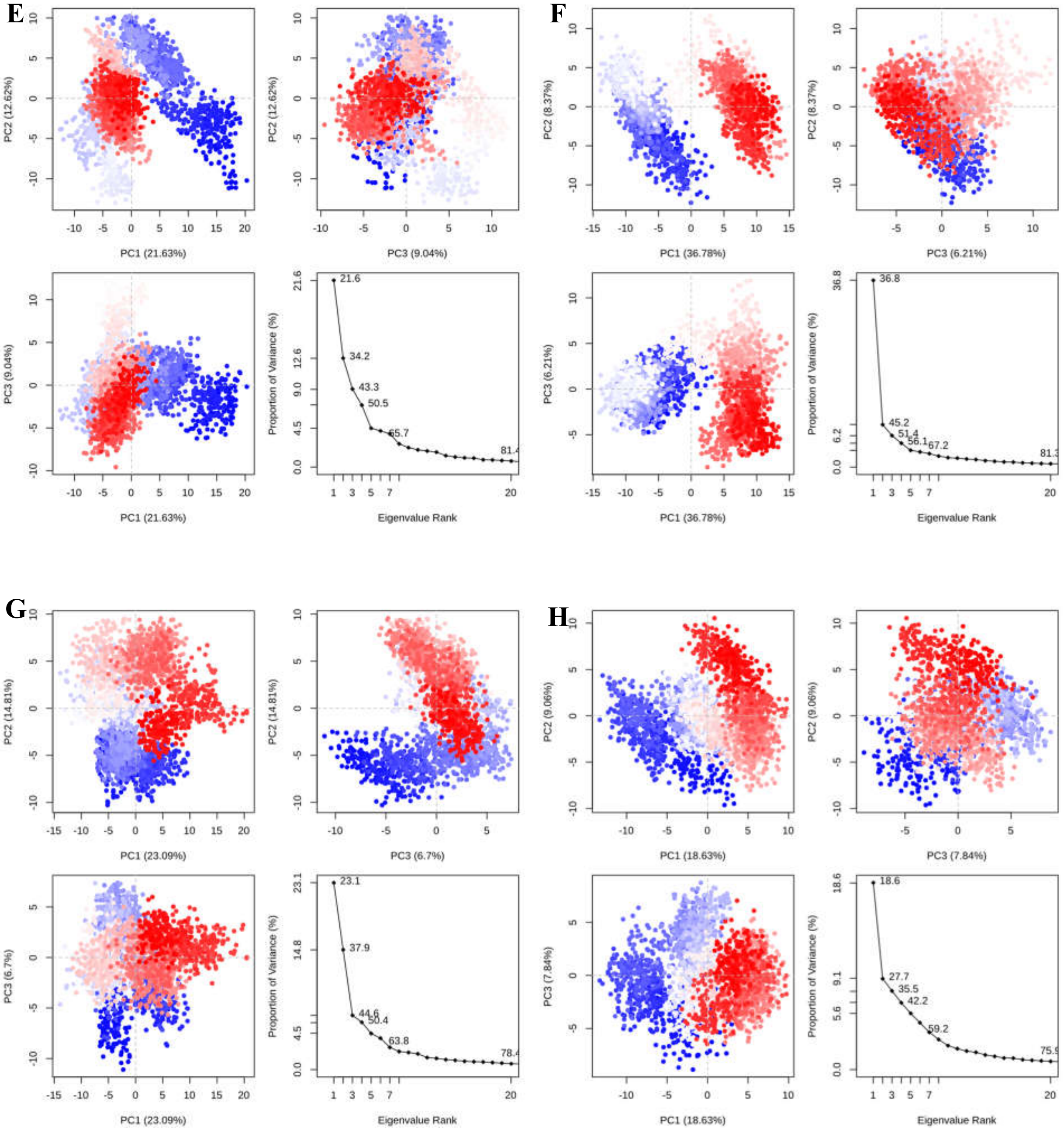

E
PC1 (21.63%)
PC2 (12.62%)
PC3 (9.04%)
Proportion of Variance (%)
Eigenvalue Rank
21.6
34.2
43.3
50.5
65.7
81.4
F
PC1 (36.78%)
PC2 (8.37%)
PC3 (6.21%)
Proportion of Variance (%)
Eigenvalue Rank
36.8
45.2
51.4
56.1
67.2
81.3
G
PC1 (23.09%)
PC2 (14.81%)
PC3 (6.7%)
Proportion of Variance (%)
Eigenvalue Rank
23.1
37.9
44.6
50.4
63.8
78.4
H
PC1 (18.63%)
PC2 (9.06%)
PC3 (7.84%)
Proportion of Variance (%)
Eigenvalue Rank
18.6
27.7
35.5
42.2
59.2
75.9

**I**

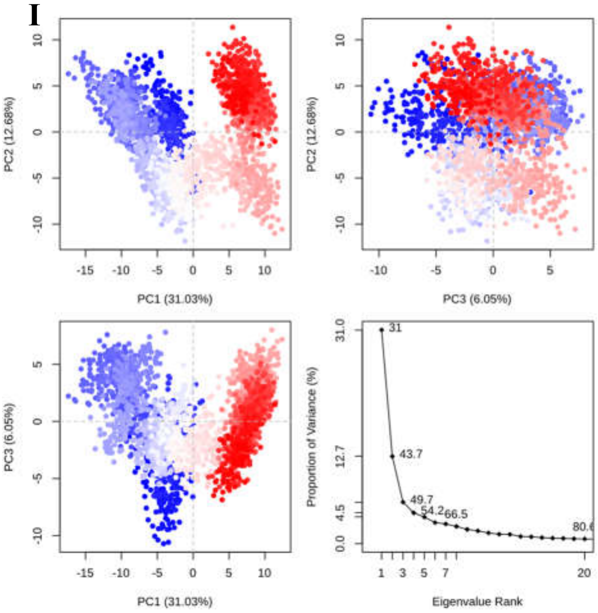


**Figure S1|** The estimation of dynamic motions of TTK complexes; **A**) TTK-NDC2 (total variance 47.34%) **B**) TTK-NDC3 (total variance 40.55%) **C**) TTK-NDC4 (total variance 49.03 %) **D**) TTK-NDC5 (total variance (44.91%) **E**) TTK-NDC6 (total variance 43.29%) **F**) TTK-NDC7 (total variance 51.26%) **G**) TTK-NDC8 (total variance 44.6%) **H**) TTK-NDC9 (total variance 35.53%) **I**) TTK-NDC10 (total variance 49.76%) The percentage of variance in three PC components of each TTK complex revealed that the protein was stable when it was bound to newly designed compounds.

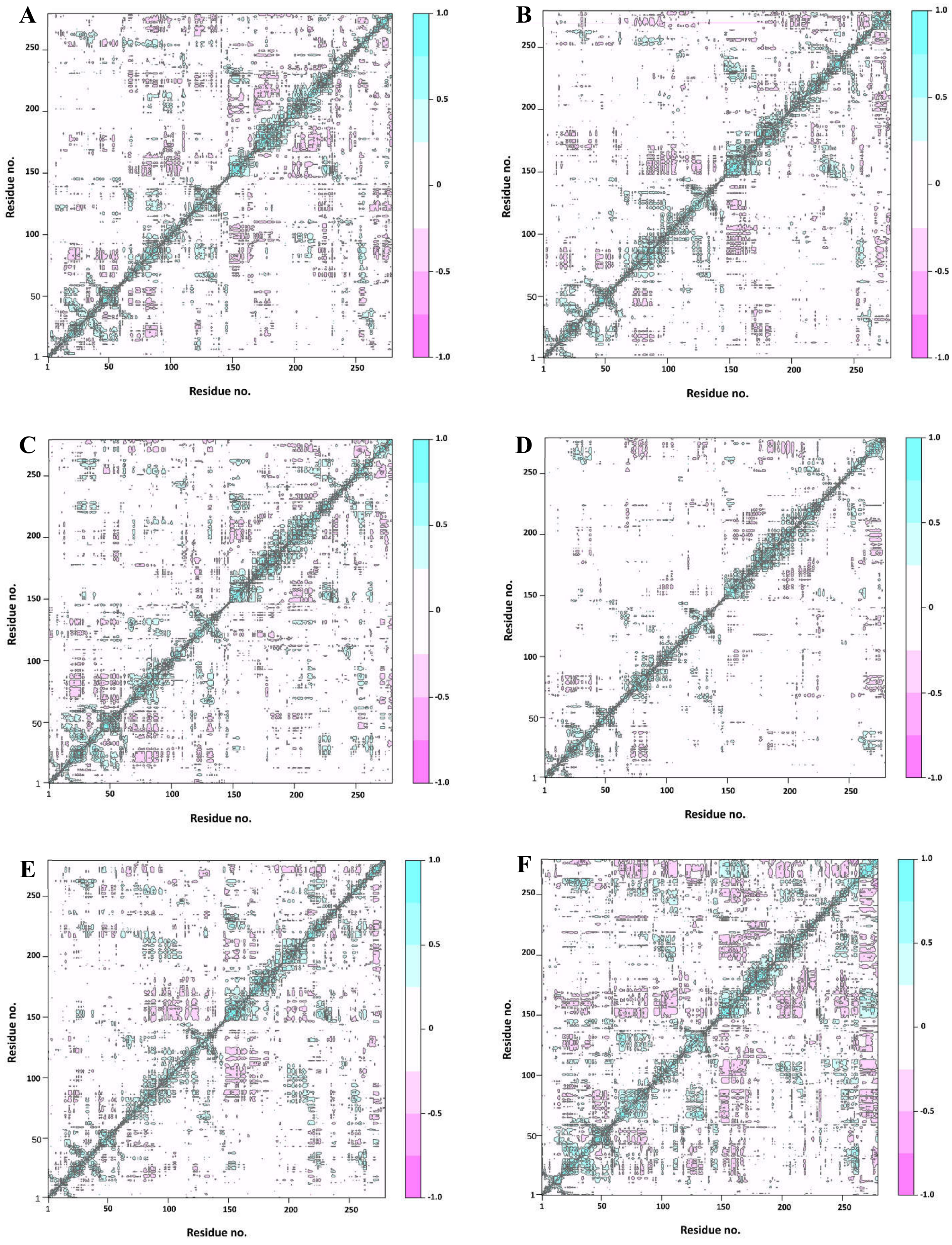
A
B
C
D
E
F
Residue no.
1.0
0.5
0
-0.5
-1.0

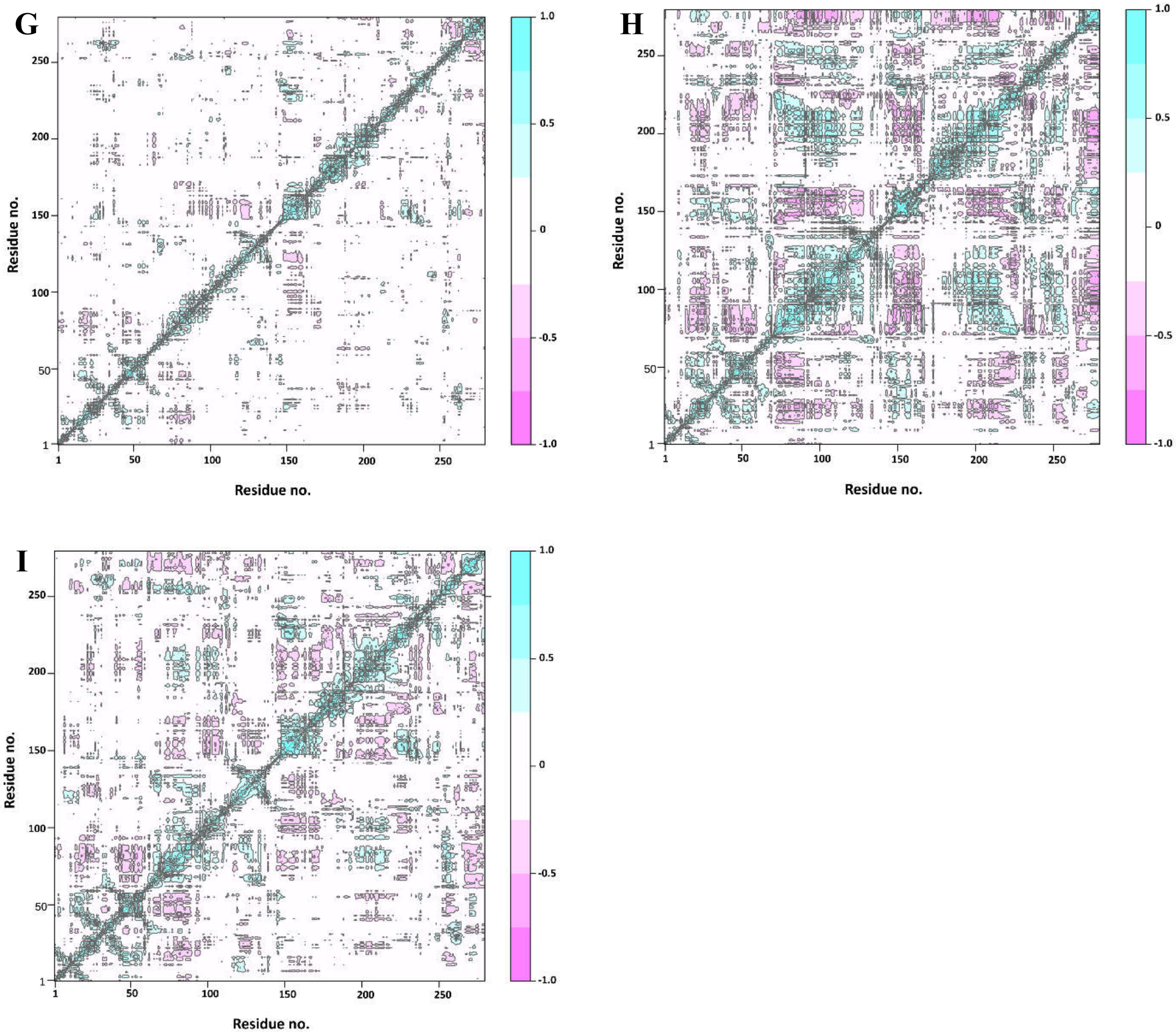


**Figure S2|** Cross correlation matrix of the TTK complexes; **A**) TTK-NDC2 **B**) TTK-NDC3 **C**) TTK-NDC4 **D**) TTK-NDC5 **E**) TTK-NDC6 **F**) TTK-NDC7 **G**) TTK-NDC8 **H**) TTK-NDC9 **I**) TTK-NDC10. Each matric showed the positive correlation motions of amino acids residues which indicated that protein remained stable when it was bound to the newly designed compounds.
